# Assessing Voice Hearing in Trauma Spectrum Disorders: A Comparison of Two Measures and a Review of the Literature

**DOI:** 10.3389/fpsyt.2019.01011

**Published:** 2020-02-24

**Authors:** Ann K. Shinn, Jonathan D. Wolff, Melissa Hwang, Lauren A. M. Lebois, Mathew A. Robinson, Sherry R. Winternitz, Dost Öngür, Kerry J. Ressler, Milissa L. Kaufman

**Affiliations:** ^1^ Psychotic Disorders Division, McLean Hospital, Belmont, MA, United States; ^2^ Department of Psychiatry, Harvard Medical School, Boston, MA, United States; ^3^ Dissociative Disorders and Trauma Research Program, McLean Hospital, Belmont, MA, United States; ^4^ Neurobiology of Fear Laboratory, McLean Hospital, Belmont, MA, United States

**Keywords:** voice hearing, auditory hallucinations, posttraumatic stress disorder, dissociative disorders, psychosis, phenomenology, assessment

## Abstract

Voice hearing (VH) can occur in trauma spectrum disorders (TSD) such as posttraumatic stress disorder (PTSD) and dissociative disorders. However, previous estimates of VH among individuals with TSD vary widely. In this study, we sought to better characterize the rate and phenomenology of VH in a sample of 70 women with TSD related to childhood abuse who were receiving care in a specialized trauma program. We compared the rate of VH within our sample using two different measures: 1) the auditory hallucination (AH) item in the Structured Clinical Interview for DSM-IV-TR (SCID), and 2) the thirteen questions involving VH in the Multidimensional Inventory of Dissociation (MID), a self-report questionnaire that comprehensively assesses pathological dissociation. We found that 45.7% of our sample met threshold for SCID AH, while 91.4% met criteria for MID VH. Receiver operating characteristics (ROC) analyses showed that while SCID AH and MID VH items have greater than chance agreement, the strength of agreement is only moderate, suggesting that SCID and MID VH items measure related but not identical constructs. Thirty-two patients met criteria for both SCID AH and at least one MID VH item (“unequivocal VH”), 32 for at least one MID VH item but not SCID AH (“ambiguous VH”), and 6 met criteria for neither (“unequivocal non-VH”). Relative to the ambiguous VH group, the unequivocal VH group had higher dissociation scores for child voices, and higher mean frequencies for child voices and Schneiderian voices. Our findings suggest that VH in women with TSD related to childhood abuse is common, but that the rate of VH depends on how the question is asked. We review prior studies examining AH and/or VH in TSD, focusing on the measures used to ascertain these experiences, and conclude that our two estimates are consistent with previous studies that used comparable instruments and patient samples. Our results add to growing evidence that VH—an experience typically considered psychotic or psychotic-like—is not equivalent to having a psychotic disorder. Instruments that assess VH apart from psychotic disorders and that capture their multidimensional nature may improve identification of VH, especially among patients with non-psychotic disorders.

## Introduction

Voice hearing (VH) is the experience of hearing voices in the absence of corresponding external stimuli. VH is typically considered a hallucinatory experience and is most frequently associated with disorders on the schizophrenia spectrum. However, VH is not specific to schizophrenia or related psychotic disorders (1–3). ‘Psychosis’ is a broad term referring to some degree of loss of contact with reality. Psychosis includes sensory experiences of things that are not physically present or beliefs that are not based in reality, among other experiences. Experiencing psychosis *symptoms* is not necessarily equivalent to having a psychotic *disorder*. Indeed, though VH is typically considered a psychotic symptom, VH occurs in many non-psychotic conditions [e.g., mood (4, 5), trauma-related (6–10), personality (11, 12), substance-related, and neurological (13–15) disorders], as well as in ‘healthy' individuals (16–21).

Trauma is a common experience. Up to 89.7% of adults have experienced at least one lifetime traumatic event (22), and up to 38.8% of the general population have experienced adversity in childhood (23). It is well-documented that trauma, and especially childhood trauma, increases the risk of both psychotic disorders and psychosis, including hallucinatory experiences [e.g., Read et al. (24) for review]. A meta-analysis aggregating data from almost 80,000 patients estimated an odds ratio of 2.78 (95% CI 2.34-3.31) for the association between childhood adversity and psychosis (25). This meta-analysis included studies that measured psychosis as a dimensional outcome (e.g., psychotic symptoms, including subclinical psychotic experiences, in the general population) as well as studies with diagnostic measures of psychosis (i.e., psychotic disorders such as schizophrenia). In fact, studies have shown a dose-response effect between cumulative childhood adversities and risk of psychosis (26–29) and hallucinations in specific (30–32). In one study, the odds of auditory hallucinations were 2.4 after one, 9.1 after two, and 7.6 after three adverse events (32). In particular, rape and physical abuse before age 16 were found to be associated with a 6-fold and 4-fold increase in auditory hallucinations, respectively (32).

Posttraumatic stress disorder (PTSD) and dissociative identity disorder (DID) are considered trauma spectrum disorders, or disorders strongly linked to trauma exposure. PTSD is characterized by persistent intrusions (e.g., flashbacks, nightmares), avoidance of trauma related stimuli, mood and cognitive alterations, and hyperarousal following exposure to one or more traumatic events (33). DID is generally conceptualized as a post-traumatic syndrome that is etiologically related to severe trauma in childhood (34, 35). DID is characterized by the presence of two or more personality states, resulting in discontinuities of experience, sense of self, and self-agency (33). Identity states are believed to reflect disruptions in the psychological integration of traumatic and non-traumatic aspects of self (36), and symptoms include gaps in memory, depersonalization, derealization, and dissociative intrusions. While DID is technically not included in the Diagnostic and Statistical Manual of Mental Disorders 5^th^ Revision (DSM-5) chapter on trauma and stressor-related disorders, there is substantial evidence that complex dissociative disorders like DID and dissociative disorder not otherwise specified (DDNOS; now called Other Specified Dissociative Disorder in the DSM-5) are post-traumatic responses to childhood trauma. Studies that have systematically examined the etiology of DID have found antecedent severe, chronic childhood trauma in almost every individual with this diagnosis (37, 38). Dissociative disorders are therefore described in the DSM-5 chapter that immediately follows the trauma and stressor-related disorders chapter, acknowledging the close relationship between DID and traumatic antecedents. Given this foundational body of work, we use the term trauma spectrum disorders (TSD) in this paper to refer to both PTSD and DID.

Multiple reports have documented the occurrence of VH in both PTSD (7, 39–48) (**Table 1A**) and dissociative disorders (8–10, 34, 49–60) (**Table 1B**). According to an early study of auditory hallucinations in combat-related chronic PTSD, voices were frequently related to trauma experienced during combat (e.g., voices of soldiers the veteran had killed or witnessed the death of), could be heard inside or outside the head, frequently commanded self-harm, were often crying or criticizing, and were relatively refractory to treatments (39). In another study of VH among chronic PTSD patients, half recognized the identity of the voices, a third experienced voices that commanded self-harm, 89% identified their voices as being inside their head, and half reported voices being as clear and loud as the researcher's voice (46). Consistent with prior studies (41, 91), the authors found that the content of voices was often symbolically or thematically related to trauma but that the voices were distinct from representations in memory of experienced events, such as flashbacks (46). The few studies that have directly compared the phenomenology of VH in trauma spectrum vs. schizophrenia spectrum disorders, though limited by small sample sizes, have shown that patients with TSD experience voices that are as frequent (6, 92), and as vivid and real as in schizophrenia (6). Similarly, loudness, negative content, and/or distress due to voices have been reported to be similar (92) or even more severe (6, 93) in TSD compared to schizophrenia.

**Table 1A:** Studies of voice hearing in Posttraumatic Stress Disorder (PTSD).

	Study	Size of Dx Sample	Female	Sample	Trauma history	Study Setting	Diagnostic Assessment	Voice Hearing Measure	Operational Definition/ Description of Voice Hearing	Voice Hearing Rate
1	Mueser and Butler (39)	N = 36	0%	Combat veterans with PTSD (mean age 38.8 years) who showed no psychotic symptoms other than AH (in n = 5)	Combat, adulthood	Inpatient unit at a VA Medical Center in California, USA	DSM-III criteria and MMPI (61)	“Clinical interviews”	Not specified (but abstract and intro briefly relate AH to “intrusive auditory perceptions,” possibly analogous to intrusive images)	13.9% AH
2	Wilcox et al (40)	N = 59	0%	Combat veterans with PTSD, consecutively treated at clinic in a 3-month period (mean age of sample not specified)	Combat, adulthood	VA outpatient clinic in Texas, USA	DSM-III criteria	Not specified	Not specified (but abstract mentions “intrusive auditory perceptions”)	28.9% life prevalence of AH
3	Butler et al (41)	N = 20	0%	38 Vietnam-era veterans, not treatment-seeking, with verifiable combat experience in the armed forces (n = 20 with PTSD, mean age 40.2 years, and n = 18 without PTSD, mean age 40.9 years). Exclusion criteria included suspected schizophrenia, psychosis, or major affective disorder.	Combat, adulthood	Veterans’ center in California, USA	DSM-III-R criteria and M-PTSD	SAPS hallucinations subscale	SAPS hallucinations subscale assesses for AH (“Have you heard voices or other sounds when no one is around?”), voices commenting (“Have you ever heard voices commenting on what you are thinking or doing?”), voices conversing (“Have you heard two or more voices talking with each other?”), somatic or tactile hallucinations, olfactory hallucinations, visual hallucinations, and a global rating of hallucination severity. Study authors summed individual subscale items (not including global ratings).	25.0% with mild-moderate hallucinations (prevalence of individual hallucination items, including AH or first rank VH, not specified)
4	David et al (42)	N = 53	0%	Veterans with chronic combat-related PTSD (mean age 46.9y) consecutively admitted	Combat, adulthood	Inpatient PTSD rehab unit in Florida, USA	SCID for DSM-III-R and M-PTSD	SCID for DSM-III-R	SCID DSM-III-R B35: “Did you ever hear things that other people couldn't hear, such as noises, or the voices of people whispering or talking? (Were you awake at the time?)” Per study authors, hallucinations were rated as psychotic if not accompanied by the perception of being back in the traumatic situation/flashbacks and if there was at least momentary disturbance in reality testing.	37.7% AH
5	Hamner et al (43)	N = 45	0%	Vietnam combat veterans with PTSD but without a primary psychotic disorder seeking treatment in a PTSD clinic (n = 22 with psychotic features, mean age 52.5 years, and n = 23 without psychotic features, mean age 51.1 years). Patients were considered to have psychotic features if they scored ≥4 (moderate or higher severity) on one of the four critical positive items on the PANSS (delusions, conceptual disorganization, hallucinatory behavior, suspiciousness/persecution).	Combat, adulthood	Outpatient PTSD clinic in South Carolina, USA	CAPS and SCID for DSM-III-R	SCID for DSM-III-R psychosis screening module and PANSS	SCID DSM-III-R B35 (see above). Per study authors, symptoms occurring only during a flashback or dissociative episode were not counted as psychotic features.	46.7% AH
6	Scott et al (44)	N = 20	95%	66 adolescents (13–18 years) consecutively admitted over 6-month study period (20 with PTSD, 18 with psychotic disorder, 28 with other disorders)	Limited details (sexual abuse in at least five patients with AH)	Adolescent inpatient unit in Brisbane, Australia	K-SADS	K-SADS items related to the form and content of hallucinations	“Has there ever been a time when you heard voices that other people could not hear?”(62)	85.0% AH (35.0% with first rank VH)
7a	Brewin and Patel (45), Study 1	N = 114	5%	Military veterans (mean age 36.3 years) receiving pensions for PTSD (93 with current PTSD, 21 with past PTSD)	Combat, adulthood	UK Service Personnel and Veterans Agency; and an ex-servicemen's mental welfare charity	SCID for DSM-IV	DES, a 28-item self-report instrument to screen for dissociative disorders	DES Q27: “Some people find that they sometimes hear voices inside their head that tell them to do things or comment on things that they are doing. Select a number that shows what percentage of the time this happens to you.”(63) Authors acknowledge it is not possible to know whether DES Q27 reflects true AH, pseudohallucinations, or some other experience.	59.6% VH
7b	Brewin and Patel (45), Study 2	N = 30	53%	30 patients (mean age 40.7 years) with PTSD arising primarily from adulthood trauma (50% also reported history of childhood trauma) were compared with trauma controls (n = 13) and depressed patients (n = 39).	Adulthood, 50% also reported childhood trauma in addition to adult trauma. Limited details on type of trauma.	Specialized PTSD clinic in London, UK	DSM-IV criteria for PTSD, confirmed by PSS	Semi-structured interview developed by the authors	Individuals were first asked about the presence of repetitive thoughts in the past week (“a stream of thoughts that repeats a very similar message over and over again inside your head”), and if so whether the individual experienced this as a voice vs. a stream of thoughts	67.0% repetitive thoughts in the form of VH. “All patients who heard voices regarded them as manifestations of their own thoughts (i.e., as pseudohallucinations)”
8	Anketell et al (46)	N = 40	7.5%	Patients with chronic PTSD (mean age 45.2 years) who underwent treatment in outpatient center	Various: serious accident/fire/explosion (82.5%), natural disaster (5%), nonsexual assault (77.5%), sexual assault (15%), sexual contact in childhood (17.5%), combat (72.5%), torture (42.5%), imprisonment (50%), life-threatening illness (20%), other traumatic events (57.5%). No distinction made between childhood versus adult trauma except for childhood sexual contact.	Psychiatric hospital outpatients and outpatient clinic in Belfast, Ireland	PDS (a 49-item self-report measure of DSM-IV PTSD)	PANSS	PANSS P3: “verbal report or behavior indicating perceptions which are not generated by external stimuli. These may occur in the auditory visual, olfactory, or somatic realms.”(64) For AH, patients were asked, “Sometimes people tell me they can hear noises or voices inside their head that others can't hear, what about you?”	50.0% current VH
9	Nygaard et al (47)	N = 181	42.5%	Trauma-affected refugees (mean age 44.9 years) with PTSD with (n = 74 PTSD-SP) and without (n = 107 PTSD) secondary psychotic features	Torture (63.5% PTSD-SP, 36.4% PTSD), imprisonment (59.5% PTSD-SP, 35.5% PTSD), lived in a war zone (85.1% PTSD-SP, 82.2% PTSD), lived in a refugee camp (25.6% PTSD-SP, 24.3% PTSD), soldier in war (31.1% PTSD-SP, 21.5% PTSD)	Specialized psychiatric unit and treatment center in the Capital Region, Denmark	ICD-10 criteria	Psychiatric records	Definition of AH not explicitly specified, but patients with PTSD with secondary psychotic features (PTSD-SP) defined: 1) psychotic symptoms had to be experienced while awake. 2) Experiences described in relation to sleep (hypnogogic/hypnopompic) not included. 3) Patients with intact reality testing included. (4) Patients with flashbacks connected to psychotic or psychotic-like symptoms were included in the study.	27.1% AH
10	Crompton et al (7)	N = 61	0%	Israeli male veterans from the 1973 Yom Kippur War. Ex-prisoners of war (POW) (mean age 53 years) with PTSD (n = 61) were compared with ex-POW's without PTSD (n = 36) and veteran controls (no POW status) without PTSD (n = 96)	Trauma related to being POW, e.g., solitary confinement, torture (sexual and physical abuse; deprivation of food, water, and medical treatment), verbal and psychological abuse. Captivity ranged from 1.5 to 8 months.	Veterans in Israel contacted by telephone and asked to take part in a longitudinal study (65)	PTSD-I	SCL-90-R	SCL-90-R Q16: “In the past week, how much were you bothered by hearing words that others could not hear?”(66)	9.8% AH at time 1 (18 years post-war); 36.2% AH at time 2 (30 years post-war)
11	Clifford et al (48)	N = 40	Not specified	Adult survivors of physical and sexual trauma with chronic PTSD (n = 40, mean age 34.4 years) were compared with healthy control participants (n = 39, mean age 29.0 years) with no history of psychiatric disorders	Physical and sexual abuse. 47.5% reported trauma in both childhood and adulthood; 52% experienced trauma only in adulthood.	Sexual assault referral center in Paddington, London, UK (n = 15) or from a database of approximately 2,000 community volunteers recruited via local newspaper advertisements, maintained by the Medical Research Council Cognition and Brain Science research group at the University of Cambridge, UK (n = 25).	SCID for DSM-IV	“Auditory pseudo-hallucinations interview” used in Study 2 by Brewin and Patel (45)(45), and DES-II	Individuals were first asked about the presence of repetitive thoughts, and if so whether the individual experienced this as a voice vs. a stream of thoughts DES-II Q27: “Some people sometimes find that they hear voices inside their head that tell them to do things or comment on things that they are doing. Circle the number to show what percentage of the time this happens to you.”(67)	5.0% “auditory pseudo-hallucinations” (32.5% VH >10% of the time on DES-II Q27, self-report) (45.0% VH >10% of the time on DES-II Q27, semi-structured interview)

**Table 1B:** Studies of voice hearing in Dissociative Identity Disorder (DID) and Other Dissociative Disorders.

	Study	Size of Dx Sample	Female	Sample	Trauma history	Study Setting	Diagnostic Assessment	Voice Hearing Measure	Operational Definition/Description of Voice Hearing	Voice Hearing Rate
1	Bliss (49)	N = 11	100%	Patients with multiple personalities (mean age 30 years) who were identified by relatives or friends	Little to no details on trauma history (one patient may have been raped)	14 individuals with multiple personalities that the author—a physician in an academic psychiatry department in Salt Lake City, Utah, USA—encountered and studied in the previous year.	“All had personalities who revealed themselves and their missions under hypnosis.” No other criteria for diagnosis specified.	713-item self-report questionnaire developed by the author (completed by only 11 of the 14 total patients)	Not specified	64% “schizophrenia” voices
2	Bliss (50)	N = 70	68.6%	Adult patients with multiple personalities (mean age not specified)	Sexual and physical abuse “when young” (60% of female and 27% of male patients reported early sexual abuse; 40% of female and 32% of male patients reported early physical abuse).	Research setting unspecified; however, author with affiliation with an academic psychiatry department in Salt Lake City, Utah, USA.	DSM-III criteria for MPD (n=20 females, n=12 males), or determined under hypnosis to have “possible multiples” even if DSM-III criteria not fully met (n=28 females, 10 males). NB: As there were no significant clinical differences observed between the DSM-III and possible multiples patients, the two groups were combined for analysis.	327-item self-report questionnaire developed by the author (consisting of items from the Research Diagnostic Criteria, the MMPI, the literature, and other sources)	Not specified	51.4% AH (54% in females, 45% in males)
3	Putnam et al (51)	N = 100	92%	Outpatients (mean age 35.8 years) identified by their treating clinician to have a diagnosis of MPD	Childhood trauma: sexual abuse (83%), physical abuse (75%), extreme neglect (60%), witness to violent death (~42%), extreme poverty (20%), and “other abuses” (~37%).	Questionnaires distributed to ~400 clinicians across North America who previously indicated an interest in MPD. Each clinician was asked to report on a single patient, currently or recently in treatment with that clinician, who met DSM-III criteria for MPD. Return rate was 40%. 100 cases were selected based on the number of questions completed (submitted by 92 clinicians, including 49 psychiatrists, 37 PhD clinical psychologists, and 6 MSW psychiatric social workers).	DSM-III criteria for MPD (criteria were specified on the cover page of the questionnaire, but “the interpretation of these criteria was left to the reporting clinician, since no independent confirmation was possible.”)	386-item questionnaire developed by the authors, including a 73-item checklist of signs and symptoms observed by the clinician or reported by the patient during clinician's initial contacts with the patient	Not specified	30% AH
4	Coons and Milstein (52)	N = 20	85%	20 patients with MPD (mean age 29 years) diagnosed by the first author, compared with 20 age and sex-matched inpatients with primarily affective and characterologic disturbances and without schizophrenia or dissociative disorders	Childhood sexual (75%) and physical abuse (55%). Childhood abuse and/or rape confirmed by at least one family member or emergency room report in 17 patients (85%)	Patients with MPD receiving treatment at an academically affiliated psychiatric hospital in Indianapolis, IN, USA (n = 10 outpatient, n = 9 inpatient, n = 1 from the consultation-liaison service) who were seen by the first author during an 11-year period.	DSM-III criteria for MPD	Psychiatric interview (unstructured) and MMPI	Not specified	60% AH
5	Kluft (53)	N = 30	70%	Patients with MPD (mean age not specified) who were determined to have achieved and maintained (for 27 months or more) fusion of personalities. Of 241 MPD interviewed patients, 135 were excluded for not having achieved fusion of personalities, 54 for not maintaining fusion 27 months or more, 11 for “relapse phenomena,” 4 for requiring psychiatric medications, and 7 for other reasons, leaving 30 patients in the study group.	Not specified	Referrals to the author—a physician affiliated with a psychiatric hospital in Philadelphia, PA, USA—for consideration or confirmation of the diagnosis of MPD.	DSM-III criteria for MPD	Data from the author's first interview with each patient	Author conducted interviews using Mellor’s first rank symptom definitions(68) (including for audible thoughts) and “generally employ[ing]” interview questions from Carpenter et al(69), with modifications. NB: the author “evolved novel questions” for voices arguing and voices conversing (exact questions not specified)	46.7% first-rank AH (33.3% voices arguing, 30% voices conversing)
6	Coons et al (54)	N = 50	92%	The first 50 patients with MPD (mean age 29 years) who were consecutively evaluated by the first author as part of a longitudinal 10-year follow-up study of patients with dissociative disorders	Childhood—sexual abuse (68%), physical abuse (60%), neglect (22%), abandonment (20%), emotional abuse (10%), witness to accidental death (4%). Adult trauma also occurred after diagnosis of MPD: rape (24%), domestic violence (10%).	Longitudinal study of MPD based in an academically affiliated psychiatric hospital in Indianapolis, IN, USA (n = 40 inpatients; n = 10 outpatients).	DSM-III criteria for MPD, and MMPI. (RDC used to diagnose 20 patients early in the study)	Modified 406-item version of the Putnam etal (51) questionnaire(51), BPRS, and DES at study intake	Which measure was used to estimate VH prevalence not specified	72% AH
7	Ross et al (55)	N = 236	87.7%	Patients with MPD (mean age 30.1 years) reported on by clinicians throughout North America via a mail-in questionnaire	Childhood sexual abuse (79.2%), childhood physical abuse (74.9%). 53.6% of the male and 67.1% of the female patients had experienced both childhood sexual and physical abuse. 63.9% of the male and 66.6% of the female patients had been raped.	Questionnaire about MPD mailed to 1,729 members of the Canadian Psychiatric Association and to 515 members of the International Society for the Study of Multiple Personality and Dissociation. Each respondent was asked to complete the questionnaire on a recent case of MPD the respondent had seen, or to indicate that the respondent had not made a diagnosis of MPD. The 236 MPD cases were reported by 203 clinicians throughout North America (including psychiatrists, non-psychiatric MD’s, psychologists, social workers, nurses, occupational therapists, and “other”)	36-item questionnaire on MPD, which inquired how well the patients met DSM-III-R and NIMH† diagnostic criteria for MPD	36-item questionnaire on MPD, which inquired about the number of Schneiderian first rank symptoms of schizophrenia experienced by the patient, among other questions	Schneiderian first rank VH (actual item(s) not specified or provided)	71.7% voices arguing, 66.1% voices commenting
8	Ross et al (56)	N = 102	90.2%	Patients with clinical diagnoses of MPD (mean age 31.8 years)	Childhood sexual (90.2%) and physical abuse (82.4%). Altogether, 95.1% of patients had experienced one or both forms of childhood trauma.	Four medical centers in North America: Winnipeg (n = 50; patients presenting for initial assessment), Utah (n = 20; patients participating as subjects for a PhD thesis), California (n = 17; patients presenting for initial assessment), and Ottawa (n = 15; patients drawn from an existing caseload, primarily therapy outpatients). No differences between centers on demographics or mean number of Schneiderian symptoms endorsed.	Clinical interview and DDIS (131-item structured interview that takes 30–45min to administer; sensitivity 90% and specificity 100% for MPD diagnosis(70, 71)	DDIS section on secondary features of MPD Note: in a complementary paper by Ross et al (1990) (72), the DDIS section on positive symptoms of schizophrenia (Schneiderian first rank symptoms) is used to estimate VH in the same sample of 102 MPD patients	DDIS Q96: “Do you hear voices talking to you sometimes or talking inside your head?” DDIS Q64: “Have you ever experienced the following: a) voices arguing in your head, b) voices commenting on your actions…” (DDIS also asks if Schneiderian symptoms have occurred only under the influence of drugs or alcohol, or only during a major depressive episode.)	87.3% voices talking (Q96) 78.4% voices arguing, 81.4% voices commenting(Q64)(72)
9	Loewenstein and Putnam (34)	N = 21	0%	21 male patients with MPD (mean age 38.6 years) were compared with data on 92 female patients previously collected for a different study on MPD (which used questionnaires mailed to outpatient clinicians across North America)(51).	Childhood—sexual abuse (85%), physical abuse (~95%), neglect (~66%), extreme poverty (~34% male), witness to violence (~40% male), other trauma (93% male, 85% female), i.e. confinement, emotional abuse, etc.	Referrals for dissociative disorder consultation to the two authors. Most patients came from the clinical services of a Veterans Affairs Medical Center in West Los Angeles, CA (patients of RJL), or from a federally operated psychiatric hospital in Washington, DC (patients of FWP), USA, although it is noted that several patients also came from other private and public settings.	DSM-III/DSM-III-R and NIMH† research criteria for MPD	386-item NIMH questionnaire (clinician-rated) describing characteristics of the patients(51)	Not specified	75% AH (vs. 30% in females)
10	Dell and Eisenhower (57)	N = 11	64%	Adolescents with MPD (mean age 14.7 years) followed in therapy by the first author	Childhood – sexual abuse (73%), physical abuse (73%), emotional abuse (82%), medical/surgical trauma (9%), injury (9%). Mean number of different types of trauma (i.e. sexual, physical, and emotional abuse, injury, medical/surgical trauma) reported was 2.4 (range, 1 to 4). Trauma and abuse was confirmed in 73% of the cases.	Referrals for diagnostic evaluation and/or therapy to the first author, a clinical psychologist affiliated with an academic medical center in Norfolk, VA, USA, over a 4-year period	DSM-III-R criteria plus NIMH† research criteria for MPD	Clinical interviews involving patients and their families	Not specified	81.8% “AH (voices heard within the head)”
11	Hornstein and Putnam (58)	N = 64	65.6%	Children and adolescents with dissociative disorders (n = 44 MPD, mean age 11.1 years; n = 20 DDNOS, mean age 8.4 years)	Childhood – sexual abuse (about 57% DDNOS, 80% MPD), physical abuse (about 64% DDNOS, 69% MPD), both sexual and physical abuse (about 50% DDNOS, 61% MPD), witnessed violence (about 80% DDNOS, 70% MPD), neglect (ab0ut 63% DDNOS, 80% MPD), abandonment (about 50% DDNOS, 46%MPD), witnessed parental death (about 12% DDNOS, 4% MPD).	First series, collected by the first author, consisted of patients (n = 22 MPD, n=8 DDNOS) seen for evaluation and treatment in an inpatient unit (except four outpatients) at UCLA in Los Angeles, CA, USA. Second series, collected by the second author, consisted of mostly outpatients (n = 22 MPD, n = 12 DDNOS) who participated in a longitudinal research project on the psychobiological effects of sexual abuse at the NIMH or who received consultation or treatment at a children's hospital in Washington, DC, USA. Data from the two series were pooled, as there were no statistically significant between-site differences for the MPD cases or the DDNOS cases (except higher rate of learning disabilities in DDNOS cases in the second series).	DSM-III-R criteria augmented by NIMH† criteria for MPD. The DSM-III-R DDNOS diagnosis lacks specific criteria; DDNOS diagnosis was given to cases in whom there were no clear-cut alter personalities but in whom there was observable dissociation manifested by amnesias, trance states, depersonalization, rapid age regression, dissociative hallucinations, and perplexing shifts in knowledge, skills, and abilities. All cases had persistence of 3+ symptoms.	Database developed by the authors to standardize data collection between the two sites. The database consisted of more than 100 symptoms and behaviors grouped into 16 factors, one of which was hallucinations.	Not specified	AH in: -97% of MPD -75% of DDNOS Command hallucinations in: -60% of MPD -25% of DDNOS “Voices urge violence” in: -41% of MPD -5% of DDNOS
12	Boon and Draijer (59)	N = 71	95.8%	Patients with MPD (mean age 33.1 years) referred from across The Netherlands; 48 referred with diagnosis of MPD prior to the study, 23 referred for evaluation because a dissociative disorder suspected by the referring clinician.	Childhood and adult trauma. History of childhood sexual or physical abuse reported by 94.4% of patients. Specifically, 77.5% of patients experienced childhood sexual abuse and 80.3% experienced physical abuse. 42.3% of patients also reported sexual and physical abuse in adulthood (individual rates not specified)	Patients referred by 60 clinicians from across The Netherlands (76% outpatients, 14.1% inpatients, 9.9% psychiatric day hospital)	Dutch version of the SCID-D (for DSM-III-R MPD)	SCID-D for DSM-III-R MPD section on associated features, Dutch version	Not specified	94.2% voices commenting, 90.1% voices talking or arguing. (Hearing voices outside one's head in only 2.8%)
13	Sar et al (8)	N = 35	88.6%	Turkish patients with clinical diagnoses of DID (mean age 22.8 years)	Childhood trauma: physical abuse (62.9%), sexual abuse (57.1%), neglect (62.9%), emotional abuse (57.1%). 77.1% of the patients experienced one or both physical and sexual abuse. 88.6% of patients experienced at least one type of childhood trauma listed above.	Medical center (including in- and outpatient psychiatric services) at the University of Istanbul, Turkey	DSM-IV criteria for DID, and n=29 (82.9%) met criteria for MPD on the Turkish version of the DDIS (sensitivity 95% and specificity 98.3%)	Turkish versions of DDIS and DES	DDIS Q96: “Do you hear voices talking to you sometimes or talking inside your head?”	94.3% “voices talking”
14	Coons (60)	N = 25	84%	Children (n = 4 DDNOS) and adolescents (n = 11 MPD, n = 9 DDNOS, n = 1 psychogenic amnesia) with DSM-III-R dissociative disorders who were consecutively referred for diagnostic evaluation (mean age of the 4 children was 8.5 years, range 5–12 years; mean age of the 21 adolescents was 15.8 years, range 13–17 years)	Childhood sexual (76%) and physical abuse (68%). Child abuse confirmed in 8 of the 9 cases of MPD and in 12 cases of DDNOS.	Referrals (n = 10 outpatient, n = 11 inpatient) for diagnostic evaluation by a specialized dissociative disorders clinic at a psychiatric hospital in Indianapolis, IN, USA between 1984-1993	DSM-III-R criteria for MPD. DDNOS was diagnosed in cases similar to MPD that failed to meet full diagnostic criteria for MPD.	Adolescents: DES and MMPI (in inpatients) Children: CDC completed by parents	Exactly how VH was defined or measured not specified	“Inner voices” reported by: -100% of adolescents with MPD -77% of adolescents with DDNOS -50% of children with DDNOS
15	Middleton and Butler (9)	N = 62	87%	Patients with DID (mean age 31.6 years)	Childhood/adolescent sexual abuse (87%), physical abuse (85%), emotional abuse (79%). Confirmation of significant past trauma was obtained for 29% of patients via actual admissions from principal abusers, other family members, photographic evidence, and medical or police records.	Patients seen over a 5-year period (1992–1997) by either of the two authors, who were affiliated with a university and a private psychiatric hospital in Queensland, Australia. Assessments were not part of a formal research project. The clinical settings were diverse (i.e., acute hospital admissions, inpatient or outpatient referrals, medical ward consultations, civil and criminal medicolegal evaluations, etc.).	DSM-IV criteria for DID, DDIS, and DES	DDIS, DES, and clinical interviews	Exactly how VH was defined or measured not specified	98% “auditory hallucinations or pseudo-hallucinations.” Both internalized and externalized voices were experienced in the majority (at least 67%; unspecified 14%)
16	Dell (10)	N = 220	90%	Patients clinically diagnosed with DID (mean age 41 years) and undergoing active psychotherapy	Not specified	Outpatient settings (n = 161) throughout the USA and Canada; inpatient facilities (n = 57) in the USA (California, Texas, Massachusetts), Canada, and Australia; clinical setting of n = 2 unknown.	Clinically diagnosed by therapist to have DSM-IV DID. Diagnosis confirmed with SCID-D-R in a subset (n = 41).	259-item precursor of the final version of the MID (with 218 items)	See **Table 2**	95% voices commenting, 89% voices arguing

AH, auditory hallucinations; BPRS, Brief Psychiatric Rating Scale (73); CAPS, Clinician Administered PTSD Scale (74); CDC, Child Dissociation Checklist (75); DDIS, Dissociative Disorders Interview Schedule (70, 71); DDNOS, Dissociative disorder not otherwise specified; DES, Dissociative Experiences Scale (63); DES-II, Dissociative Experiences Scale, version II (67); DSM, Diagnostic and Statistical Manual of Mental Disorders [e.g., DSM-III (76), DSM-IV (77)]; K-SADS, Kiddie Schedule for Affective Disorders and Schizophrenia for School Aged Children (62); MID, Multidimensional Inventory of Dissociation (78); MMPI, Minnesota Multiphasic Personality Inventory (79); MPD, Multiple Personality Disorder; M-PTSD, Mississippi Scale for Combat-Related Posttraumatic Stress Disorder (80); PANSS, Positive and Negative Syndrome Scale (64); PDS, Posttraumatic Stress Diagnostic Scale (81); PSS, Posttraumatic Stress Scale (82); PTSD-I, Posttraumatic Stress Disorder Inventory (83); RDC, Research Diagnostic Criteria (84); SAPS, Scale for the Assessment of Positive Symptoms (85); SADS-C, Schedule for Affective Disorders and Schizophrenia, Change Version (86); SCID, Structured Clinical Interview for DSM [SCID for DSM-III-R (87), SCID for DSM-IV (88)]; SCID-D, Structured Clinical interview for DSM-III-R Dissociative Disorders (89); SCID-D-R, Structured Clinical interview for DSM-IV Dissociative Disorders-Revised (90); SCL-90-R, Symptom Checklist 90, Revised (66); VH, voice hearing.

†The National Institute of Mental Health (NIMH) research criteria for MPD specified that, in addition to DSM-III or DSM-III-R criteria for MPD being satisfied, two or more alter personalities had to exhibit distinct alter personality-specific behavior on at least three separate occasions, and that psychogenic amnesia was reported or observed(34).

While VH appears to be associated with significant morbidity in many individuals with TSD, the scope of the problem remains unclear, as estimates of VH prevalence in TSD vary widely (**Table 1A****,** **B**). Some studies report VH in as many as 85% of PTSD patients (44) and in 90% or more of individuals with DID (8–10, 58–60), suggesting that the experience of VH may potentially be the rule rather than the exception in TSD. Other studies, however, report rates as low as 5% in PTSD (48) and 30% in DID (51).

The wide range of estimates may be due, in part, to differences in the types of patients who were sampled. Rates and experiences of VH may differ according to the individual's sex, age, specific type of trauma, timing of exposure to trauma within the developmental lifespan, level of overall clinical acuity at the time of assessment, and broader cultural factors, among others. Most studies of VH in PTSD have been conducted in male combat veterans (7, 39–43, 45, 46). Fewer studies have examined VH in mostly women (44) or mixed sex civilian samples (45, 47, 48) with PTSD. (Conversely, females are disproportionately represented among individuals who are diagnosed with dissociative disorders, and this sex discrepancy is reflected in the existing literature.) Further, while exposure to severe physical and sexual abuse in early childhood have been prominent themes in the dissociative disorders literature, there are no studies examining VH in individuals with PTSD primarily related to trauma in childhood.

Studies also vary considerably with respect to how VH or hallucinations in TSD are ascertained. Numerous studies provide little to no details about how VH was defined or measured (9, 34, 39, 40, 51, 52, 54, 57, 58, 60); in some cases, the lack of specific details may be due to the fact that estimating the prevalence of VH, per se, was not many studies’ primary objective. Along similar lines, several studies, especially earlier studies, used less rigorous methods for ascertaining VH, such as unstructured interviews (39, 40, 52, 53, 57, 58), medical records review (47), or measures that have not been well-studied or validated (49, 50, 53). Among the PTSD and dissociative disorders studies that measured VH more systematically, some [e.g., (7, 50, 52)] used instruments designed to assess a broad range of psychopathology such as the Minnesota Multiphasic Personality Inventory (MMPI) (79). Others (8–10, 45, 56, 59, 60) measured VH using instruments designed to assess dissociative symptomatology, such as the Dissociative Experiences Scale (DES) (63) or DES-II (67). Still others (41–44, 46) measured auditory hallucinations using instruments designed to evaluate symptoms associated with schizophrenia spectrum disorders, such as the Positive and Negative Syndrome Scale (PANSS) (64) or the psychotic disorders module of the Structured Clinical Interview for DSM (SCID) (87).

Importantly, the nature of VH in PTSD and DID is not well understood and warrants further exploration. It is unclear if the experience of VH in trauma-related disorders reflects perceptual abnormalities as appears to be the case in psychotic disorders, hearing the voices of different identity state intrusions in DID, and/or something else entirely (10). It has been proposed that VH in non-psychotic disorders as well as in the general population may be more dissociative rather than psychotic (2, 94) and, thus, different from the perceptual abnormalities experienced by individuals with schizophrenia. On the other hand, a small literature suggests there is significant phenomenological overlap between VH experiences in schizophrenia and trauma-related disorders (6, 92, 93). Still other researchers have proposed that VH is entirely dissociative in nature (94); according to this more radical view, auditory hallucinations are a symptom of dissociation even in schizophrenia. Given the limited understanding about the phenomenology of VH in TSD, how a specific item asks about VH or auditory hallucinations—for example, how a question is worded, and the specific context in which the question is asked—is likely to be critical in influencing how an individual responds, and in determining VH prevalence.

This investigation sought to better characterize the phenomenology and rate of VH in a sample of adult female patients with PTSD related to childhood abuse and neglect who were receiving care at a specialized PTSD treatment center. We sought to estimate the proportion of individuals with VH in our sample using two different instruments—one that is traditionally used to diagnose schizophrenia and related psychotic disorders, and the other representing a model of pathological dissociation. The goal was to investigate how much the estimates of VH rate differ with the two measures, and to examine whether the measures differ in clinically or phenomenologically meaningful ways.

## Methods

### Participants and Procedures

We studied 73 women with PTSD either recruited from inpatient, residential, partial hospital, and outpatient programs specializing in the treatment of adult women with histories of trauma-related disorders at McLean Hospital (n = 68) or referred by affiliated outpatient trauma clinicians (n = 5). Patients were recruited as part of a multimodal neuroimaging study evaluating the neurobiology of traumatic dissociation. The study was approved by the institutional review board of McLean Hospital, and all participants provided written informed consent.

To be eligible for the study, participants had to be female, 18–89 years in age, have legal and mental competency, meet criteria for PTSD, and have a history of childhood abuse. Individuals meeting criteria for schizophrenia or other psychotic disorders (including bipolar I disorder with psychotic features) were excluded. Exclusion criteria also included alcohol or substance dependence or abuse within the last month, history of neurological conditions that may cause significant psychiatric symptomatology, history of head injury or loss of consciousness longer than five minutes, and delirium secondary to medical illness. As data were originally acquired for a neuroimaging study, individuals with any contraindications to magnetic resonance imaging (e.g., metal implants, pregnancy, claustrophobia, etc.) were also excluded.

We administered the Clinician Administered PTSD Scale for DSM-5 (CAPS-5) (95) to diagnose PTSD, the Structured Clinical Interview for DSM-IV Dissociative Disorders Revised (SCID-D-R) (90) to diagnose DID, the SCID for DSM-IV Axis II personality disorders (SCID-II) (96) to diagnose borderline personality disorder, and the SCID-I for DSM-IV-TR (97) to assess for any other Axis I disorders, including schizophrenia and other psychotic disorders. These four semi-structured interviews are considered the gold standard instruments for diagnosing the respective categories of psychiatric disorders. Clinical research interviews were conducted by trained research staff with expertise in trauma spectrum disorders.

In addition to diagnostic interviews, participants completed the Multidimensional Inventory of Dissociation (MID) (78), a 218-item (168 dissociation items and 50 validity items) self-report questionnaire that comprehensively assesses the phenomenological domains for pathological dissociation. The MID assesses the presence of 23 different dissociative symptoms and is unique in that it inquires about only pathological rather than normative experiences of dissociation (e.g., absorption, hypnotizability, fantasy proneness). For each item, the individual is asked to rate the frequency of experiences using a 0–10 Likert scale in which 0 indicates “never” and 10 “always.” Participants are instructed to indicate a number between 0 and 10 that “best describes how often it happens to you.” The MID explicitly instructs participants to rate how often each experience occurs when not under the influence of alcohol or drugs. The MID has two scoring systems: mean frequency scores and severe dissociation scores. Severe dissociation scores are based on empirically-determined pass/fail cut-off scores for each item, designed to maximize the discrimination between individuals who have and do not have a severe dissociative disorder (10). The instrument shows excellent internal consistency (Cronbach's alpha = 98), test-retest reliability over 4–8 weeks, construct validity with standardized measures of traumatic stress, and discriminative validity with the SCID-D-R. Structural validity has been confirmed using factor analytic techniques (78, 98).

Additional self-report measures completed by participants include the Childhood Trauma Questionnaire (CTQ) (99), a 25-item questionnaire that evaluates the frequency of childhood abuse and neglect, the PTSD Checklist for DSM-5 (PCL-5) (100, 101), a 20-item measure that assesses the twenty DSM-5 symptoms of PTSD, and the Beck Depression Inventory (BDI) (102), a 21-item scale that assesses the severity of depression. The CTQ measures the severity of five different types of childhood trauma (emotional, physical, and sexual abuse and emotional and physical neglect). To characterize the specific features of childhood trauma in finer detail, as well as to assess exposure to non-child abuse trauma, we also administered the Traumatic Events Inventory (TEI) (103), which assesses lifetime history of trauma exposure.

We obtained information about demographic characteristics, including age, parental education, and intelligence quotient (IQ) as measured by the Wechsler Abbreviated Scale of Intelligence (WASI-II) (104). We also collected information about psychiatric medications (i.e., antipsychotics, antidepressants, mood stabilizers, and sedative-hypnotics) patients were taking at the time of study participation.

We grouped the patients into one of three VH categories: unequivocal voice hearers, ambiguous voice hearers, and unequivocal non-voice hearers (described in greater detail below, in the section on “Phenomenological comparison of the unequivocal vs. ambiguous voice hearing groups”). We compared these three groups with respect to demographic and clinical characteristics. All statistical analyses for this study were conducted using SPSS version 24.0. We first evaluated variables for normality with the Shapiro-Wilk test. For continuous variables that were normally distributed, we performed one-way analysis of variance (ANOVA) to test the null hypothesis that at least one of the three groups is different. Statistically significant results from ANOVA were followed by post-hoc pairwise testing using Tukey's honestly significant difference (HSD) test to adjust for multiple comparisons. For continuous variables that did not fit a normal distribution, we conducted the non-parametric Kruskal-Wallis test. Statistically significant between-group differences detected by the Kruskal-Wallis test were followed by post-hoc pairwise comparisons using the Mann-Whitney U test, with p-values Bonferroni adjusted for three pair-wise tests. For categorical variables, we conducted chi-square tests.

### Rate of Voice Hearing in PTSD

We investigated the rate of VH in PTSD using two different measures:

1. **Item B16 (Auditory Hallucinations) in the SCID psychosis module**. This item asks, “Did you ever hear things that other people couldn't, such as noises or the voices of people whispering or talking?” Individuals who scored 3 (“threshold or true”) were considered to meet criteria for lifetime auditory hallucinations, while individuals with scores of 1 (“absent or false”) or 4 (“inadequate information”) were considered not to meet threshold for this item. No individuals reported experiences consistent with a score of 2 (“subthreshold”) in our sample. To calculate the rate of VH, we calculated the proportion of individuals who scored threshold on B16. In the DSM-IV-TR, items B17 and B18 also assess for auditory hallucinations, specifically about whether voices commented on what the individual was doing or thinking, or whether two or more voices conversed with one another, respectively. However, item B16 serves as the gatekeeper to items B17 and B18; that is, an individual must meet criteria for B16 in order to be asked about B17 and B18. As B17 and B18 data are not available for all participants, we did not further analyze these data.
2. **MID voice hearing items**. The MID contains thirteen questions that inquire about VH experiences (**Table 2**). MID Q98, which asks about voices that come from “unusual places” such as the air conditioner or computer, serves as a screen for psychosis. The other twelve items (Q6, Q30, Q42, Q84, Q97, Q118, Q140, Q159, Q171, Q199, Q207, Q216) ask about VH in the context of pathological dissociation. As mentioned above, the MID uses pass/fail cut-off scores to identify whether an experience was present to a clinically significant degree (78). For the VH items, these cut-off scores are either ≥ 1 (Q30, Q42, Q98, Q118, Q199) or ≥ 2 (Q6, Q84, Q97, Q140, Q159, Q171, Q207, Q216). A VH experience was considered to be present (dichotomous, yes or no) to a clinically significant degree if the cut-off threshold was met for that item. We calculated the rate of VH by identifying the proportion of individuals who met clinically significant criteria for one or more of the 12 MID VH experiences (exclusive of the psychosis screener, Q98).

**Table 2:** Voice hearing items in the Multidimensional Inventory of Dissociation (MID).

Item	Multidimensional Inventory of Dissociation (MID) Voice Hearing Questions	MID Voice Hearing Category				
		SCH	PER	CHI	INT	POS
Q6	“Hearing the voice of a child in your head.”			X		
Q30	“Hearing voices in your head that argue or converse with one another.”	X				
Q42	“Hearing a voice in your head that tries to tell you what to do.”	X			X	
Q84	“Hearing a voice in your head that wants you to hurt yourself.”		X			
Q97	“Hearing a lot of noise or yelling in your head.”	X		X		
Q98	“Hearing voices, which come from unusual places (for example, the air conditioner, the computer, the walls, etc.), that try to tell you what to do.”					
Q118	“Hearing voices crying in your head.”			X		
Q140	“Hearing a voice in your head that calls you names (for example, wimp, stupid, whore, slut, bitch, etc.)”	X	X			
Q159	“Hearing a voice in your head that wants you to die.”		X			
Q171	“Hearing a voice in your head that calls you a liar or tells you that certain events never happened.”	X	X			
Q199	“Hearing a voice in your head that tells you to ‘shut up'.”	X			X	
Q207	“Hearing a voice in your head that calls you no good, worthless, or a failure.”	X	X			
Q216	“Hearing a voice in your head that is soothing, helpful, or protective.”					X

SCH, Schneiderian voices; PER, Persecutory voices; CHI, Child voices; INT, Voices of internal struggle; POS, Positive voices.

Of the 73 patients in the study at the time of analysis, SCID data were missing for two participants and MID data missing for an additional participant. As the primary objective of this project was to compare VH as measured by SCID B16 versus the MID, we conducted all analyses using only data from the 70 participants for whom both SCID and MID data were available.

### Receiver Operating Characteristics (ROC) Curves

As we observed discrepant rates of VH using SCID B16 and MID VH items, we sought to examine the relative agreement between SCID B16 and MID VH measures. To do this, we used a receiver operating characteristic (ROC) curve to examine the total dissociation score for the twelve MID VH items (excluding the psychosis screen, Q98) against dichotomized SCID B16 (yes or no) as the outcome variable. The dissociation score for the twelve VH items reflects the number of pathological VH symptoms experienced by each individual, and the possible range of VH dissociation scores was 0–12.

We additionally examined total VH frequency, which takes into account the severity of individual items across the full range of the Likert scale, against SCID B16. For this ROC analysis, we took the sum of the frequency ratings across all twelve MID VH items. For items with a cut-off score of ≥2, we recoded the ratings of frequency (0-10 Likert scale) such that ratings <2 were equal to 0, a rating of 2 was equal to 1, a rating of 3 was equal to 2, and so on, such that the maximum frequency rating for each item with ≥2 cut-off was 9. Since there are eight items with cut-off ≥2 that needed to be recoded, the maximum possible sum of the pathological VH frequencies decreased from the original 120 to 112.

For both ROC curves, we calculated the area under the curve (AUC), and used a statistical significance threshold of p < 0.05 (or a 95% confidence interval) to reject the null hypothesis that the accuracy of the MID VH items in predicting VH as measured by SCID B16 is due to chance.

### Phenomenological Comparison of the Unequivocal vs. Ambiguous Voice Hearing Groups

**Figure 1:**
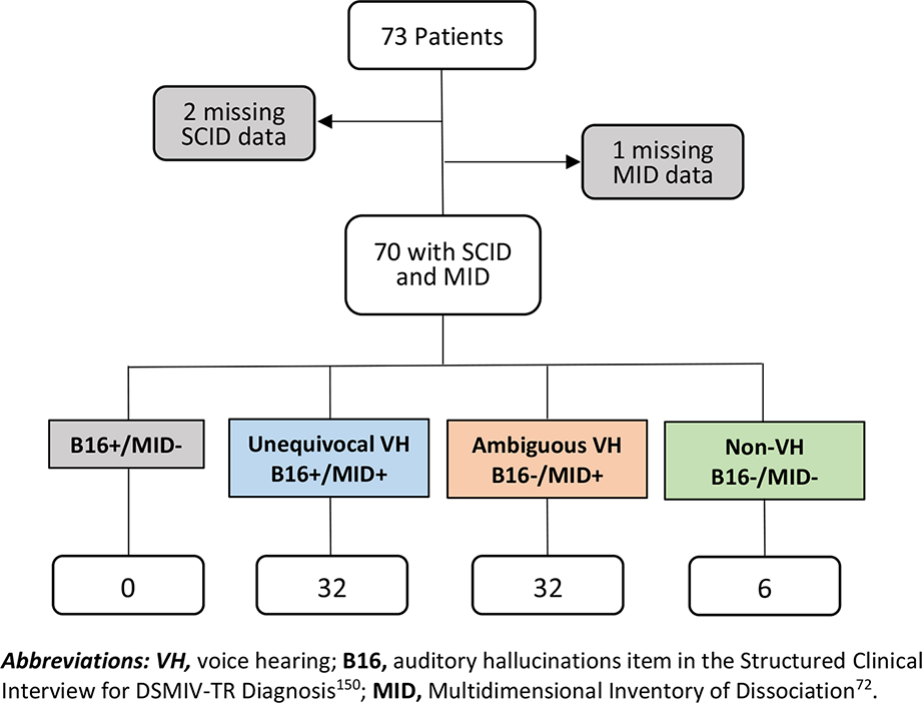
Breakdown of participants.

We sought to determine whether any phenomenological VH characteristics distinguished individuals with VH who met threshold for B16 from those who did not, i.e., whether there were any systematic differences in the quality or severity of VH experiences among individuals who endorsed any VH. To do this, we separated the patients into three different groups (**Figure 1**):

1. Trauma patients with **unequivocal VH**, i.e., those who met threshold for both SCID B16 (Auditory Hallucinations) AND one or more MID VH items (B16+/MID+),
2. Trauma patients with **ambiguous VH**, i.e., those who did not meet threshold for SCID B16 but did meet clinically significant criteria for one or more MID VH items (B16-/MID+), and
3. Trauma patients who **unequivocally denied VH**, i.e., those who did not meet threshold for VH by either measure (B16-/MID-).

There were no individuals who met B16 criteria but did not meet clinically significant criteria for MID VH (B16+/MID-).

We compared the two VH groups (unequivocal vs. ambiguous VH groups) with respect to VH phenomenology, as measured by the MID. The phenomenological categories in the MID related to VH are voices arguing (Q30), voices commenting (Q42, Q97, Q140, Q171, Q199, Q207), persecutory voices (Q84, Q140, Q159, Q171, Q207), child voices (Q6, Q97, Q118), and helper voices (Q216). Some VH items in the MID appear in more than one VH category (e.g., Q97 is included in both the Schneiderian voices and child voices categories). These MID categories were derived from standard measures of dissociative experiences, interviews with dissociative patients, and theoretical models of pathological dissociation (78). The subscales of the MID were optimized for clinical diagnostic utility, however, principal axis factor analysis in two large datasets (N's >1000) extracted 12 factors that conceptually mapped onto the clinically-optimized subscales of the MID (78). We combined the voices arguing and voices commenting items into a single Schneiderian VH category. The category called “voices/internal struggle” also contains two VH items (Q42, Q199). However, this category includes seven other items not related to VH. Moreover, Q42 and Q99 are already included in the Schneiderian VH category. Therefore, we did not further examine the voices/internal struggle category. In total, we looked at four phenomenological categories of VH: Schneiderian, persecutory, child, and helper voices.

Mirroring the two scoring systems of the MID instrument, we compared the VH phenomenology of the two VH groups using two different approaches. In the first approach, we calculated a dissociation score for each category, which is the number or count of items for which an individual meets clinical significance within a category (e.g., Schneiderian, persecutory, etc.). Whether an individual meets clinical significance for an item is a dichotomous outcome, based on the specific cut-off in the MID for that item. The higher the dissociation score, the greater the number of items in a VH category an individual has experienced. The maximum possible score for each category varies according to the number of items in each category (i.e., seven items for Schneiderian voices, five items for persecutory voices, three items for child voices, and one item for helper voices). In the second approach, we calculated the mean frequency of pathological VH, which is the mean Likert scale rating of all items within each VH category. The frequency rating reflects how often someone experiences a particular item; hence, the mean frequency reflects the average of the individual item frequency ratings within a VH category.

As the dissociation scores and mean frequencies for the four categories of pathological VH were not normally distributed, we conducted the nonparametric Mann-Whitney U test to test the null hypothesis that the unequivocal VH and ambiguous VH groups have equal severity in these VH measures. Probability values were Bonferroni-adjusted to account for our testing four different VH categories. We conducted post-hoc analyses to identify which specific item(s) within each category accounted for those between-group differences in VH categories only for categories found to be statistically significantly different between the two VH groups.

## Results

### Participant Characteristics

As a whole, the patients in this study represent a highly traumatized group. All patients had histories of childhood abuse, as this was a criterion for study inclusion. Of the 66 patients for whom TEI data about child abuse were available, 74% experienced verbal abuse during childhood; 69% were beaten or physically punished; 27% had been attacked by someone with a gun, knife, or other weapon during childhood; 65% had been attacked by someone without a weapon during childhood; and 83% were sexually abused in childhood, with 77% of patients experiencing sexual abuse before the age of 14 (**Supplementary Table 1**). Patients generally experienced abuse very early in life, starting at approximately 5 years of age on average. Furthermore, the duration of childhood abuse was often prolonged (mean duration 16.5 years for verbal abuse, 10.8 years for beatings, and 8.0 years for sexual abuse during childhood) (**Supplementary Table 2**). Perpetrators of childhood abuse (specifically verbal abuse, beatings, or sexual abuse) were a primary caregiver in 85% of cases, other family in 63%, and non-family in 65% of cases (**Supplementary Table 3**). Only 20% of patients had only one abuser during childhood; 80% reported multiple childhood abusers (mean 2.9, SD 1.5, range 1-7 for n=65 reporting child verbal abuse, beatings, or sexual abuse). Moreover, for most patients, traumatic experiences were not limited to abuse in childhood; patients reported multiple different types of trauma (relational as well as trauma related to natural disasters, serious accident or injury, and sudden life-threatening illness) during their lifetimes (mean 8.4, SD 3.6, range 2-18 for n=67 for whom any TEI data were available) (**Supplementary Table 4**). Notably, none of the patients in this sample had trauma from military service or combat.

The three groups—trauma patients with unequivocal VH (B16+/MID+), trauma patients with ambiguous VH (B16-/MID+), and trauma patients who unequivocally denied VH (B16-/MID-)—were comparable with respect to age, IQ, parental education, co-occurring major depressive disorder, co-occurring borderline personality disorder, severity of depressive symptoms, severity of PTSD symptoms, and medication exposure at the time of the study (**Table 3**).

**Table 3:** Participant characteristics.

	All Patients	Unequivocal VH	Ambiguous VH	Unequivocal non-VH	Statistic	Significance
**Sample Size**	**70**	**32**	**32**	**6**		
**Primary Trauma-Related Diagnosis**					χ^2^ = 8.860	*p* = 0.065
PTSD without dissociation, No. (%)	16 (22.9%)	4 (12.5%)	8 (25.0%)	4 (66.7%)	**χ^2^ = 8.561**	***p* = 0.014**
PTSD Dissociative Subtype, No. (%)	17 (24.3%)	8 (25.0%)	8 (25.0%)	1 (16.7%)	χ^2^ = 0.207	*p* = 0.902
PTSD + Major Dissociative Disorders	37 (52.9%)	20 (62.5%)	16 (50.0%)	1 (16.7%)	χ^2^ = 4.453	*p* = 0.108
PTSD + DDNOS, No. (%)	3 (4.3%)	0 (0.0%)	3 (9.4%)	0 (0.0%)	–	–
PTSD + DID, No. (%)	34 (48.6%)	20 (62.5%)	13 (40.6%)	1 (16.7%)	–	–
**Co-occurring Disorders**							
Current Major Depressive Disorder	5 (7.1%)	3 (9.4%)	1 (3.1%)	1 (16.7%)	χ^2^ = 1.840	*p* = 0.399
Borderline Personality Disorder	15 (21.4%)	6 (18.8%)	7 (21.9%)	2 (33.3%)	χ^2^ = 0.645	*p* = 0.724
**Age** ^1^ **, median (range), y**	31 (18–62)	31.5 (18–61)	30 (18–60)	32 (21–62)	χ^2^ = 0.665	*p* = 0.717
**IQ, mean ± SD** ^2^	118.3 ± 8.6	118.0 ± 8.4	119.0 ± 8.7	116.7 ± 9.9	*F* = 0.201	*p* = 0.819
**Parental Education, No. (%)** ^3^	35 (51.5%)	16 (51.6%)	16 (51.6%)	3 (50.0%)	χ^2^ = 0.006	*p* = 0.997
**Childhood Trauma Questionnaire (CTQ)** ^1,4^ **, median (range)**	79 (38–119)*	86 (27–119)^*^	75 (38–113)	62.5 (52–79)	χ^2^ = 3.875	*p* = 0.144
Emotional Abuse	18 (6–25)	18 (5–25)	18 (8–25)	17 (11–19)	χ^2^ = 1.568	*p* = 0.457
Physical Abuse	10 (5–25)	12 (5–23)	10 (5–25)	8 (5–19)	χ^2^ = 1.184	*p* = 0.553
Sexual Abuse	21 (5–25)*	21 (5–25)^*,^^†^	17 (5–25)	10 (5–22)^†^	**χ^2^ = 6.218**	***p* = 0.045**
Emotional Neglect	18 (5–25)	19 (7–25)	17 (5–25)	14.5 (7–24)	χ^2^ = 1.034	*p* = 0.596
Physical Neglect	12 (5–21)	12 (5–21)	12 (5–21)	10 (6–13)	χ^2^ = 1.002	*p* = 0.606
**MID Severe Dissociation Score, mean ± SD**	89.3 ± 41.3	99.6 ± 43.8^†^	85.2 ± 38.0	56.0 ± 24.2^†^	***F* = 3.315**	***p* = 0.042**
**PTSD Checklist for DSM-5 (PCL-5) Score, mean ± SD**	52.3 ± 13.6	50.0 ± 16.1	55.2 ± 9.1	49.0 ± 18.4	*F* = 1.355	*p* = 0.265
**Beck Depression Inventory (BDI), mean ± SD**	29.0 ± 10.8	32.7 ± 11.5	27.9 ± 10.9	25.8 ± 6.8	*F* = 1.886	*p* = 0.160
**Medication** ^5^						
Antipsychotics, No. (%)	30 (47.6%)	14 (43.8%)	11 (34.4%)	5 (83.3%)	χ^2^ = 3.853	*p* = 0.146
Antidepressants, No. (%)	46 (73.0%)	19 (59.4%)	21 (65.6%)	6 (100.0%)	χ^2^ = 3.101	*p* = 0.212
Mood Stabilizers, No. (%)	25 (39.7%)	12 (37.5%)	10 (31.3%)	3 (50.0%)	χ^2^ = 0.486	*p* = 0.784
Sedative-Hypnotics, No. (%)	33 (52.4%)	15 (46.9%)	15 (46.9%)	3 (50.0%)	χ^2^ = 0.035	*p* = 0.983
**Race**					χ^2^ = 8.344	*p* = 0.401
White, No. (%)	61 (87.1%)	30 (93.8%)	25 (78.1%)	6 (100%)	–	–
Black, No. (%)	3 (4.3%)	1 (3.1%)	2 (6.3%)	0 (0%)	–	–
Asian, No. (%)	4 (5.7%)	0 (0%)	4 (12.5%)	0 (0%)	–	–
American Indian, No. (%)	1 (1.4%)	0 (0%)	1 (3.1%)	0 (0%)	–	-
Other, No. (%)	1 (1.4%)	1 (3.1%)	0 (0%)	0 (0%)	–	–
**Ethnicity**					χ^2^ = 3.591	*p* = 0.464
Hispanic, No. (%)	2 (2.9%)	0 (0%)	2 (6.2%)	0 (0%)	–	–
Non-Hispanic, No. (%)	67 (95.7%)	31 (96.9%)	30 (93.8%)	6 (100%)	–	–
Prefer not to answer, No. (%)	1 (1.4%)	1 (3.1%)	0 (0%)	0 (0%)	–	–

^1^Age and CTQ scores were not normally distributed, thus the Kruskal-Wallis test was conducted. ^2^Eight participants are missing WASI IQ scores (4 unequivocal VH+; 4 ambiguous VH+). ^3^At least one parent with a 4-year college degree; two participants are missing information on parental education level (1 unequivocal VH+; 1 ambiguous VH+). ^4^Two participants are missing CTQ scores (1 unequivocal VH+; 1 ambiguous VH+). ^5^Seven participants are missing information on medication (3 unequivocal VH+; 4 ambiguous VH+). ^*^One participant declined to answer questions from the Sexual Abuse subscale of the CTQ. The statistical tests, including the mean and standard deviation, of the Sexual Abuse subscale and the total CTQ score are reflective of this. ^†^No significant difference was detected between unequivocal VH+ and ambiguous VH+ groups in the post hoc test.

Statistics in which at least one of the three groups is significantly different (p < 0.05) are shown in bold.

When we assessed for any differences in trauma-related diagnoses between the three VH groups, we observed a trend for the unequivocal non-VH group to be characterized by a higher proportion of individuals with PTSD alone (i.e., individuals with neither a dissociative subtype of PTSD nor a diagnosis of a major dissociative disorder in addition to PTSD) (*p* = 0.065). To further explore the possibility that the three VH groups might be confounded by different trauma-related diagnoses, we performed a separate post-hoc analysis for each PTSD subgroup (i.e., PTSD alone, PTSD dissociative subtype, and PTSD plus major dissociative disorder). We found a statistically significant association between VH group and primary diagnosis for the subgroup of 16 patients who had PTSD alone (*p* = 0.014), with the majority of these patients (66.7%) unequivocally reporting no VH, and fewer of these individuals having either unequivocal (12.5%) or ambiguous VH (25.0%). There were no associations between VH group and primary diagnosis for the subgroups of patients with PTSD dissociative subtype (*p* = 0.902) or PTSD with a major dissociative disorder (*p* = 0.108). In spite of the significant association between the non-VH group and the diagnosis of PTSD without dissociation, as mentioned, the three VH groups did not differ with respect to severity of PTSD symptoms, as measured by the PCL-5.

Consistent with the above, ANOVA showed that at least one of the three VH groups was statistically significantly different with respect to the severe dissociation score for the full MID (*F_2,67_* = 3.315, *p* = 0.042). Post-hoc pair-wise analysis showed that the statistically significant difference was driven by the difference between the unequivocal VH and unequivocal non-VH groups (*p* = 0.044). The unequivocal non-VH group had a significantly lower MID severe dissociation score compared to the unequivocal VH group. The ambiguous VH group was comparable with both the unequivocal VH group (*p* = 0.325) and the unequivocal non-VH group (*p* = 0.236) in severe dissociation score.

Furthermore, though the three groups reported similar overall severity of childhood trauma as measured by the CTQ total score, and comparable CTQ subscale scores for emotional abuse, physical abuse, emotional neglect, and physical neglect in childhood (**Table 3**), the groups were significantly different in severity of childhood sexual abuse (*p* = 0.045*)*. Again, post-hoc pair-wise comparisons showed that the group-wise difference in CTQ childhood sexual abuse was due to a difference between the unequivocal VH and unequivocal non-VH groups (*p* = 0.045, Bonferroni-adjusted for three tests), with the unequivocal non-VH group showing significantly lower mean childhood sexual abuse score. Importantly, there were no statistically significant differences in childhood sexual abuse between the two VH groups (*p* = 0.510, Bonferroni-adjusted).

With respect to specific types of trauma exposures, the three VH groups did not significantly differ on exposure to the various types of child abuse or overall lifetime trauma assessed by the TEI (**Supplementary Tables 1** and **4**). The groups also showed no differences with respect to who perpetrated the abuse experienced during childhood (**Supplementary Table 3**). However, there was a significant difference in the age at which patients first experienced physical abuse, with both the unequivocal and ambiguous VH groups reporting earlier first ages of physical abuse compared to the non-VH group. No differences were seen in the first age at which verbal and sexual abuse began (**Supplementary Table 2**).

### Rates of Voice Hearing in PTSD: SCID vs. MID

**Figure 2:**
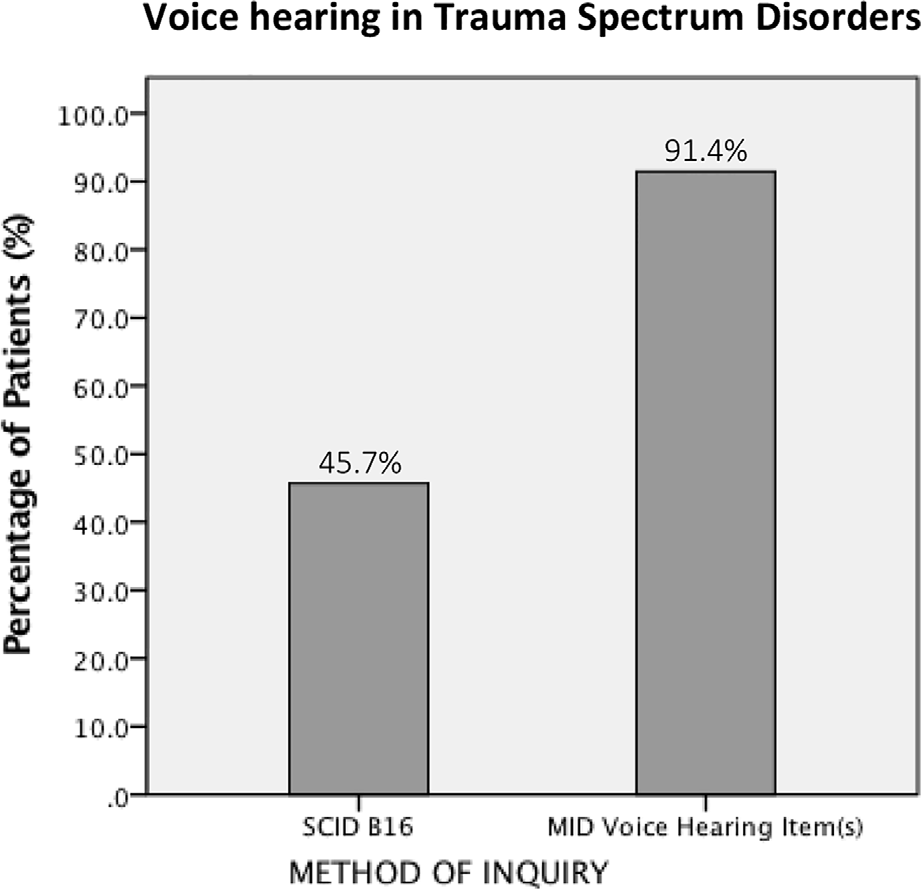
The rate of voice hearing in PTSD as assessed by two different measures.

Thirty-two (45.7%) of the 70 total patients included in the analyses met threshold for SCID item B16 (Auditory Hallucinations). When we calculated the VH rate using the MID, i.e., examining the proportion of patients meeting clinically significant criteria for at least one MID VH item, the rate went up to 91.4% (64 of 70 patients) (**Figure 2**).

### ROC Curves

**Figure 3:**
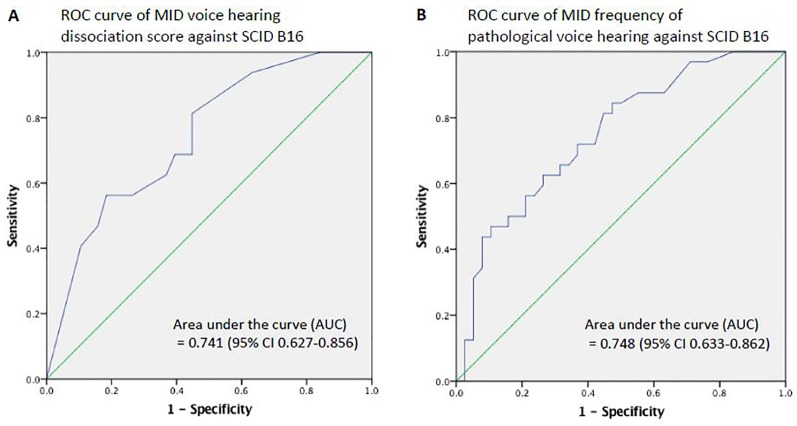
Receiver operating characteristics (ROC) curves. **(A)** The MID voice hearing dissociation score reflects the total number of pathological VH items in the MID experienced by each individual (possible range 0-12). **(B)** MID voice hearing frequency is the sum of severity scores of individual items across the full range of the Likert scale (possible range 0-112). While B16 and MID VH scores have greater than chance agreement, the strength of agreement is only moderate, suggesting that B16 and MID VH items measure related, but not identical, constructs.

We conducted ROC analyses to assess the ability of the MID VH items to predict an endorsement of auditory hallucinations as measured by SCID B16. We calculated the ROC curves using two slightly different summary measures for MID VH. When we investigated the MID VH dissociation score against B16, the AUC was 0.741 (95% CI 0.627-0.856, *p* = 0.001) (**Figure 3A**). When we investigated the MID frequency of pathological VH against B16, the AUC was 0.748 (95% CI 0.633-0.862, *p* = 3.87 x 10^-4^) (**Figure 3B**).

### Phenomenological Comparison of the Unequivocal vs. Ambiguous Voice Hearing Groups

**Figure 4:**
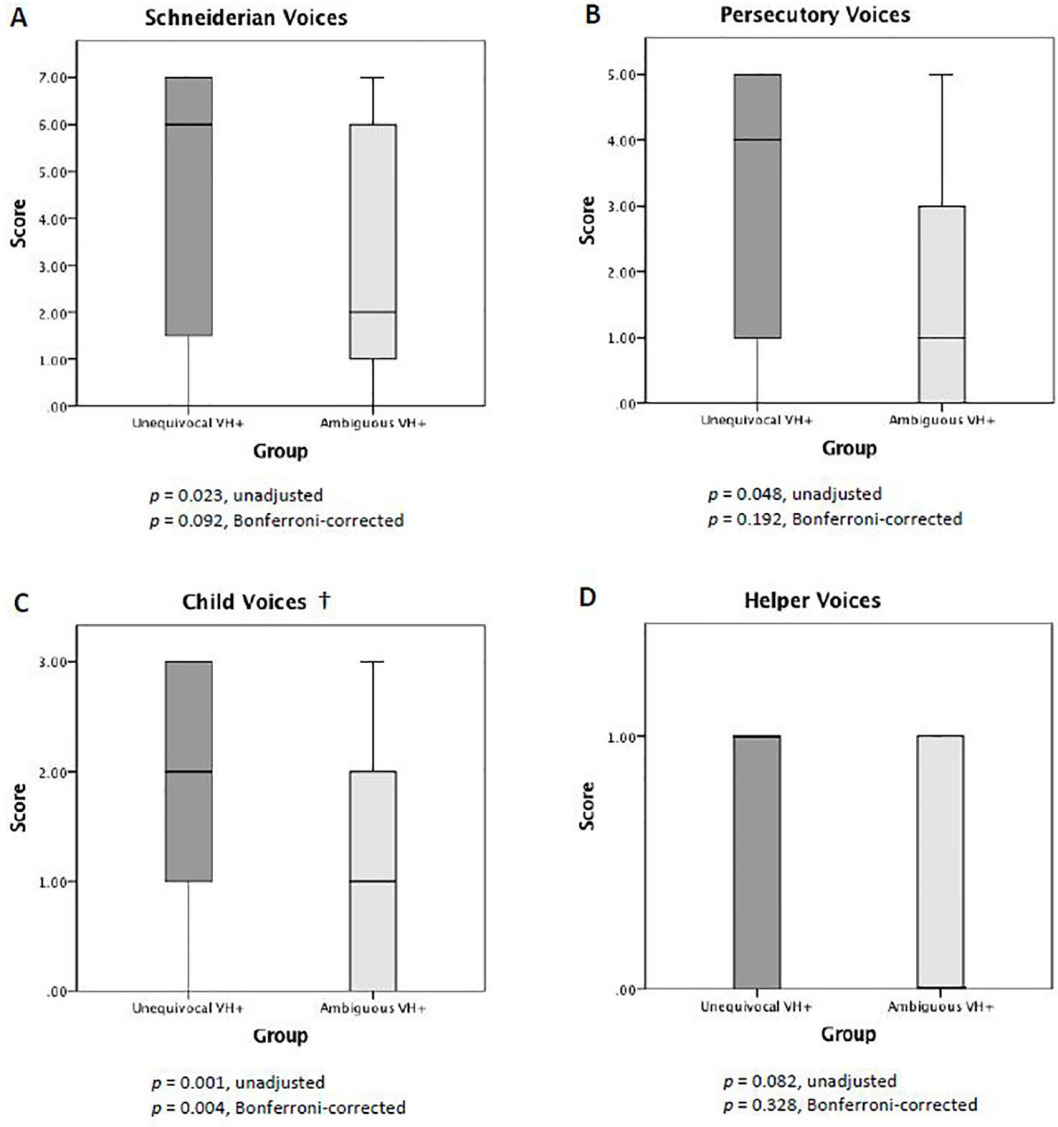
Scores for pathological voice hearing. Compared to the ambiguous VH group, the unequivocal VH group showed higher MID VH dissociation scores for Schneiderian voices **(A)**, persecutory voices **(B)**, and child voices **(C)**. †Only the between-group difference in child voices **(C)** survived multiple comparisons correction.

The two VH groups showed systematic differences in VH phenomenology. When we examined between-group differences in dissociation score for each VH scale, we found the unequivocal VH group to have higher scores for Schneiderian voices, persecutory voices, and child voices relative to the ambiguous VH group. However, only the between-group difference in child voices survived Bonferroni-correction for multiple comparisons (**Figure 4**). Post-hoc analysis of specific items in the child voices scale revealed that a significantly greater proportion of individuals in the unequivocal vs. ambiguous VH groups endorsed Q97 (“hearing a lot of noise or yelling in your head”) (71.9% vs. 34.4%; *p* = 0.009, Bonferroni-corrected) and Q118 (“hearing voices crying in your head”) (62.5% vs. 21.9%, *p* = 0.003, Bonferroni-corrected). There was no between-group difference in Q6 (“hearing the voice of a child in your head”) (56.3% vs. 34.4%; *p* = 0.237, Bonferroni-corrected), which is the other item in the child voices scale.

**Figure 5:**
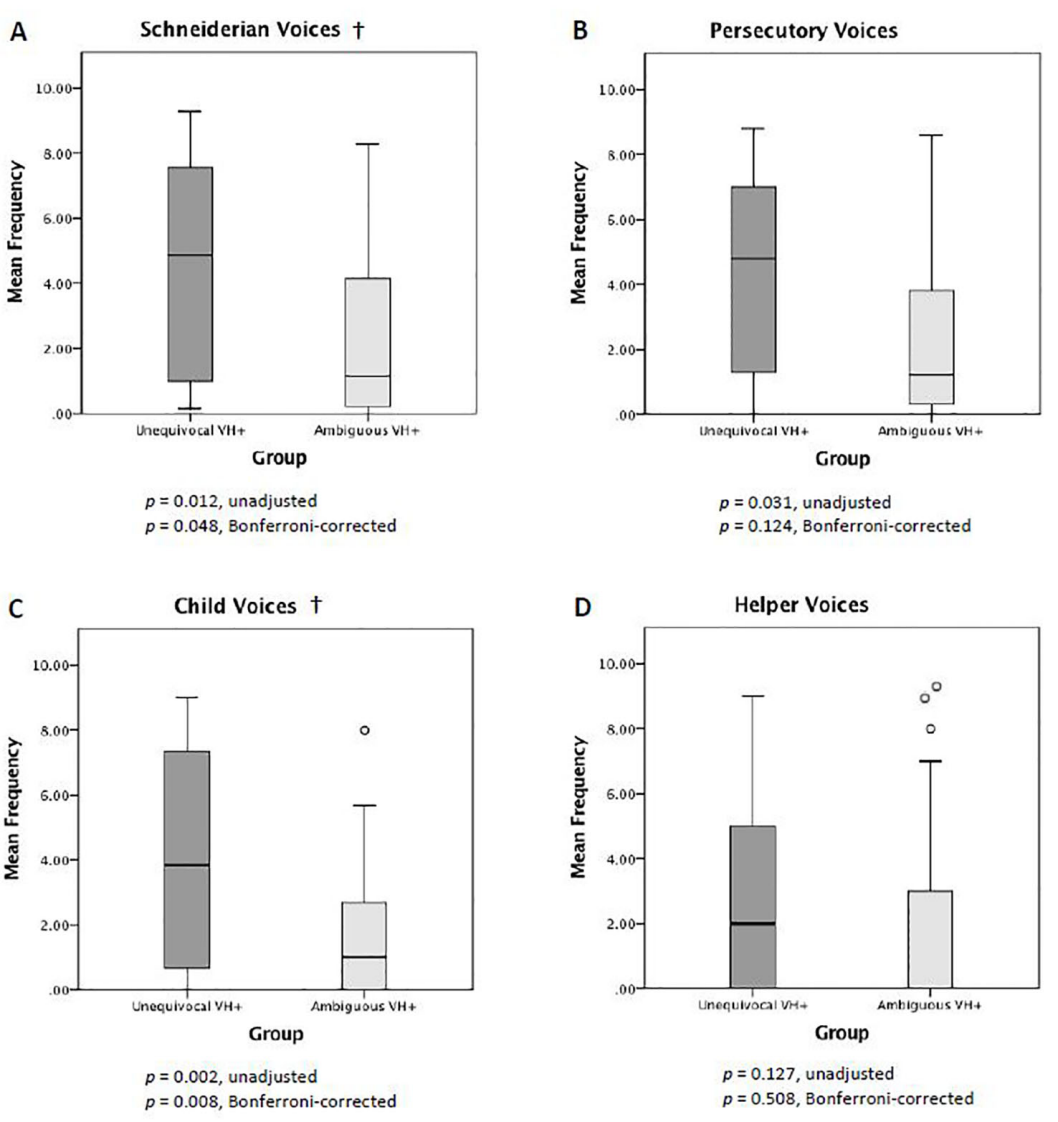
Mean frequency of pathological voice hearing. Compared to the ambiguous VH group, the unequivocal VH group showed higher mean MID VH frequency scores for Schneiderian voices **(A)**, persecutory voices **(B)**, and child voices **(C)**. †The between-group differences in Schneiderian voices **(A)** and child voices **(C)** survived multiple comparisons correction.

When we examined between-group differences in mean frequency for each VH scale, we found the unequivocal VH group to have higher mean frequencies for Schneiderian voices, persecutory voices, and child voices. Only the differences in Schneiderian voices and child voices survived Bonferroni correction (**Figure 5**). When we performed post-hoc analyses of individual items in these two scales, we found that the unequivocal VH group, relative to the ambiguous VH group, reported higher frequencies for items Q42 (“hearing a voice in your head that tries to tell you what to do”) (median 4 vs. 0, *p* = 0.027, Bonferroni-corrected), Q97 (5 vs. 0, *p* = 0.018, Bonferroni-corrected), and Q118 (3.5 vs. 0, *p* = 0.009, Bonferroni-corrected). We found no statistically significant differences between unequivocal and ambiguous VH groups in Schneiderian VH items Q30 (6 vs. 0, *p* = 0.054), Q140 (6.5 vs. 1, *p* = 0.684), Q171 (1 vs. 0, *p* = 1), Q199 (4 vs. 0, *p* = 1), Q207 (7 vs. 2, *p* = 0.612) (NB: all p-values are Bonferroni-corrected for 9 comparisons). Again, among the child voices items, there was no between-group difference in item Q6 (3.5 vs. 0, *p* = 0.234, Bonferroni-corrected).

## Discussion

In this study, we estimated the proportion of patients with VH in a sample of female patients with PTSD and dissociative disorders related to a history of childhood abuse and neglect. We estimated this using two different measures—item B16 in the SCID-IV-TR, the gold-standard instrument to diagnose DSM-IV-TR schizophrenia and related psychotic disorders, and the Multidimensional Inventory of Dissociation (MID), a comprehensive self-report questionnaire that assesses the phenomenological domains for pathological dissociation. We found that VH is common, regardless of method of inquiry. However, the rate of VH depends on how you ask. In a single patient sample, we found the proportion of individuals with VH to be 45.7% when using SCID item B16 and 91.4% when measured with the MID. ROC analyses showed that while SCID B16 and the MID VH items have greater than chance agreement, the strength of the agreement is only moderate, suggesting that B16 and the MID VH items measure related, but not identical, constructs. Finally, we found that there were systematic differences between unequivocal voice hearers (individuals who endorsed both B16 and MID VH items) and ambiguous voice hearers (those who endorsed MID VH items but not B16) in VH phenomenology.

### It Matters How You Ask

One of the main findings from our study is that how someone is asked about VH matters. The proportion of VH individuals detected by the MID was double that identified by the SCID. This is a striking discrepancy in VH rate given that both estimates arise from the same patient sample. The discrepancy can be explained by considering differences in the way that the MID and SCID B16 ascertain VH. First, the MID assesses VH through the lens of primarily dissociative experiences, while B16, which is embedded within the psychotic disorders module of the SCID, is immediately preceded by a series of questions about delusions and followed by questions about hallucinations in other sensory modalities. These other psychotic symptoms may resonate less well with patients with trauma-related and other non-psychotic disorders and make it less likely for a patient to endorse B16 even if, in a strict sense, the individual has heard “things that other people couldn't, such as noises or the voices of people whispering or talking.” Both patients and interviewers may add layers of meaning and interpretation to B16 based on the context in which B16 is asked, and patients may harbor concerns that they will be diagnosed with a psychotic disorder if they answer affirmatively to *any* item in the psychotic disorders module. For example, some participants in this study asked if B16 includes flashbacks. From DSM-III (76) through DSM-IV-TR (105), because of the special significance given to voices commenting or voices conversing in diagnosing schizophrenia, having either of those VH experiences alone without other core (criterion A) symptoms could, in fact, lead to a diagnosis of schizophrenia. According to studies that have examined pathways to care in patients with dissociative disorders, between 15.6% to 49% of individuals received a diagnosis of schizophrenia prior to being diagnosed with DID (8, 9, 51, 54, 55, 59, 72). More appropriately, the DSM-5, hearing voices commenting or conversing in isolation is no longer sufficient to meet diagnostic criteria for schizophrenia (33).

Second, the MID asks about VH experiences with thirteen different questions, and thus is more likely to capture at least one VH experience compared to the single auditory hallucination item in the SCID. While items B17 (voices conversing) and B18 (voices commenting) in the SCID also inquire about auditory hallucinations, those items are assessed only if the patient initially responds affirmatively to the B16 gatekeeper item. As such, the SCID provides only one opportunity to screen for VH.

Third, the MID utilizes a 0–10 Likert scale format and offers a wider spectrum of responses, with finer gradations based on severity/frequency, while the SCID B16 forces ratings into more limited and categorical response options (i.e., absent, subthreshold, or present, with a fourth option if there is insufficient information). Even if MID dissociation scores transform frequency ratings into a dichotomous variable based on a clinical cut-off, respondents are not aware of this when they respond, and cut-off scores vary from item to item.

Fourth, the MID is a self-report questionnaire, while the SCID is a semi-structured interview. While semi-structured interviews administered by experienced clinicians are considered the gold-standard method of conducting a psychiatric evaluation, a self-administration format may allow individuals to disclose about VH and other experiences that are considered pathological with potentially less stigma, shame, and judgment. Self-administration forms would thus be expected to increase reporting compared to a clinician administered interview, and this phenomenon has been observed in the PTSD literature [e.g., meta-analysis by (106)]. On the other hand, self-reporting could also lead to an effect in the opposite direction. A recent study (48) found that the same question about VH (DES-II item 27) resulted in endorsement by 32.5% with self-report vs. 45% during semi-structured interview, suggesting that self-report could also lead to underreporting of VH (or over-reporting in semi-structured interview).

Fifth, all the VH items in the MID—except for the psychosis screener, MID Q98—asks about VH “in your head.” It is unclear what impact the qualification of “in your head” might have on how individuals respond to the MID items. Whether VH location is a clinically significant phenomenological feature has been debated for decades. Some studies have reported that a greater proportion of trauma than schizophrenia patients experience internal voices (44, 93), but other studies that have directly compared trauma and schizophrenia spectrum patients on this feature have reported no difference (6, 12, 92, 107). Furthermore, many studies have shown that VH location does not separate cleanly by diagnosis; internal and external voices appear to be prevalent in both trauma-related and schizophrenia spectrum patients, and many individuals experience both (12, 44, 92, 93, 107). A review that found that the proportion of people with exclusively external VH and, conversely, exclusively internal VH are similar in schizophrenia, dissociative disorders, other psychiatric patients, and non-patients (94). Though voices heard inside the head have occasionally been referred to in the literature as “pseudohallucinations,” the term—which has been used variably and inconsistently to refer not only to internal voices, but also perceptual disorders lacking sensory vividness, hallucinations with intact reality-testing and insight, ego-syntonic versus alien voices, voluntary or involuntary voices, and/or isolated hallucinations without other psychopathology (108, 109)—has been argued to lack construct validity (109). In the case of MID VH items, “in your head” could refer to a voice that is heard internally rather than externally, or it could reflect that the voice hearer has insight that the experience is a product of the voice hearer's mind. How critical the phrase “in your head” is in capturing the VH experience in this patient sample is unknown, since there are no MID VH items that are phrased comparably but without the words “in your head”. Nevertheless, “in your head” may be associated with less stigma and greater acceptability, and thereby increase rates of affirmative responses to MID VH. Unlike the MID, item B16 in the SCID does not make any specifications about whether voices and other sounds must be heard outside the head for the experience to meet threshold, though this does not rule out the possibility of interviewer bias when rating B16.

Lastly, the MID and SCID B16 differ with respect to the time frame that is assessed. The MID instructs individuals to indicate “how often do you have the following experiences…,” in the present tense, suggesting a focus on current or recent experiences. The SCID, on the other hand, assesses whether the individual has “ever” in his or her lifetime had VH or other auditory hallucinatory experiences. Assessing for “ever” experience of VH would be expected to have the effect of inflating the proportion of patients endorsing B16. However, in our sample, asking about lifetime VH with B16 did not outweigh other factors, as a substantially greater proportion of patients endorsed MID VH relative to B16.

Beyond considerations about differences in the format and contextual characteristics of the instruments themselves, the data additionally provide insights into differences between the measures' ability to detect potentially substantive clinical differences. We found systematic differences between individuals who endorse both measures (unequivocal voice hearers) and those who endorse VH only on the MID (ambiguous voice hearers). The unequivocal VH group reported significantly higher scores and mean frequencies on the child voices scale, and higher mean frequencies on the Schneiderian voices scale, compared to the ambiguous VH group. When we examined the individual items within the child voices and Schneiderian voices scales, hearing voices with the phenomenological qualities of crying, commanding, or voices that sound like a lot of noise or yelling in the head most distinguished unequivocal from ambiguous voice hearers. These differences in the two VH groups do not appear to be due merely to differences in clinical severity, as there were no statistically significant differences between the unequivocal and ambiguous VH groups on demographic or clinical characteristics, including on measures of childhood sexual abuse, depression severity, PTSD symptom severity, or severity of dissociation.

### Psychosis, Dissociation, and/or Other?

In this paper, we analyzed VH using two measures intended to assess different constructs: SCID B16, which measures schizophrenia-related auditory hallucinations, and the MID, which measures pathological dissociation. Thus, we interpret our data primarily through these lenses, of psychosis and dissociation, respectively.

The German psychiatrist Kurt Schneider described specific forms of VH—consisting of two or more voices conversing with one another, or voices commenting on one's thoughts, beliefs, and actions—as “first rank,” or pathognomonic of schizophrenia (110). Reflecting this historical conceptualization, schizophrenia and related disorders are the only major psychiatric disorder category in the current Diagnostic and Statistical Manual of Mental Disorders (DSM-5) (33)—other than substance related disorders, neurocognitive disorders (e.g., Lewy body disease), or delirium—that explicitly includes hallucinations in the diagnostic criteria. Despite the fact that VH is not uncommon in trauma spectrum disorders, neither hallucinations nor VH are included in the diagnostic criteria for PTSD or DID. In the DSM, there is no mention of VH in the criteria or text description of PTSD. For DID, while the DSM-5 text description of diagnostic features mentions that individuals with DID can experience “perceptions of voices (e.g., a child's voice; crying; the voice of a spiritual being),” and that in some cases, “voices are experienced as multiple, perplexing, independent thought streams over which the individual experiences no control” (p. 293) (33), VH and other passive-influence experiences are not part of the DSM DID diagnostic criteria (10). Thus, when clinicians encounter a patient who reports hearing voices in the absence of substances and non-psychiatric medical or neurological conditions, they are often faced with the dilemma of whether to diagnose that individual with a psychotic disorder, even when such a diagnosis may not otherwise fit with the overall clinical presentation. VH frequently leads to a diagnosis of primary psychotic disorder when, in fact, underlying trauma-related disorders may sometimes be primary.

The results of our ROC curve analyses suggest that VH measured by the MID and auditory hallucinations measured SCID B16 are related but not identical constructs. While the exact pathophysiology underlying VH remains unknown, different meanings, mechanisms, and conceptual frameworks have been proposed for VH in schizophrenia spectrum vs. TSD. Auditory hallucinations in schizophrenia and related psychotic disorders are commonly conceptualized as a problem of over-perceptualization [e.g., (111–115)] as well of impaired reality monitoring and misattribution [e.g., (116, 117)], i.e., inner speech and thoughts that are internally generated are perceived as originating outside the self. Importantly, evidence suggests that VH may be a consequence of faulty circuitry, or dysconnectivity, in the brain (118). Studies implicate disruptions both within and between brain areas involved in auditory perception, speech and language, top-down inhibition, memory encoding and/or storage, self-monitoring, and error prediction, among other brain functions (112, 115, 119–124).

In the context of TSD, VH is more often conceptualized within a psychological framework, i.e., as a psychological reaction to traumatic or emotionally overwhelming events to which the individual has previously been exposed (125). Voices commenting and voices conversing—along with other forms of “made” actions, feelings, and impulses—are considered to reflect dissociative intrusions, which are intrusions into consciousness by a self-state that is partially dissociated (10). When chronically exposed to traumatic events, integrative capacities become compromised and traumatic experiences are not appropriately encoded and consolidated in autobiographical memory (126). The personality becomes divided, with traumatic memories less accessible to autobiographical narrative memory (126). VH, while distinct from flashbacks, is one form in which such dissociated traumatic content can be reactivated into conscious awareness (2, 125). These conceptual frameworks, relating to psychotic vs. dissociative VH, highlight different mechanisms, but the models are not necessarily mutually exclusive, and VH in schizophrenia and TSD may share areas of overlap.

The MID is designed to detect pathological dissociative experiences and 91.4% of patients met criteria for at least one MID VH item. Thus, it seems clear that VH has some element of dissociation for the majority of individuals in our sample. Our finding that women with PTSD who endorsed both B16 and MID VH items had significantly greater and more severe experiences on the child voices scale compared to those who endorsed VH only on the MID can be interpreted within the context of dissociation. Child voices in individuals with trauma-related disorders who report histories of childhood abuse and neglect are conceptualized as compartmentalized intrusion symptoms, and may represent reexperience of past traumatic events (127). In DID, such intrusions often are personified as “child voices” and are relationally significant. Child voices may represent cognitions, affects, or conflicts held in relationship to significant attachment figures from childhood (128). While child voices are considered in this context within a dissociation model of VH, it is possible that the dissociative experience of hearing child voices, especially child voices crying or yelling, is experienced as more severe, intrusive, and alien than other dissociative experiences, and that this is what leads to a greater likelihood for an individual to meet threshold for B16. On the other hand, we also found that individuals who endorsed both B16 and MID VH items also reported greater severity of Schneiderian voices. Schneiderian symptoms, which are symptoms relating to a breakdown of self-other boundaries, have traditionally been considered to belong in the realm of psychosis. Thus, it is also possible to interpret VH experiences in the unequivocal VH group in the context of psychosis.

Unfortunately, our data are limited in the ability to determine whether the VH experiences in the subset of individuals who endorse SCID B16 in addition to MID also have a psychotic component similar to auditory hallucinations in schizophrenia spectrum disorders. In our ROC curve analyses, we set SCID B16 as the reference measure, as VH is usually considered a hallucinatory experience and the SCID psychotic disorders module is the gold standard for identifying psychotic symptoms in the context of diagnosing psychotic disorders. By doing so, we make the assumption in our ROC analysis that B16 represents “true” or a more accurate measure of VH. However, given the relatively limited research in this area, we do not know if B16 functions the same in individuals with trauma spectrum disorders as it does in psychotic disorders. In our trauma spectrum sample, it is unclear if B16 is actually detecting psychosis, merely a more severe form of dissociation, and/or something else. Accuracy can be defined in terms of both sensitivity (the ability of a test to correctly identify true positives) and specificity (the ability of a test to correctly identify true negatives). As the MID detects more individuals with VH experiences, it could potentially be argued that the MID is a more sensitive measure. Conversely, if SCID B16 identifies VH experiences that are more psychotic in nature, then B16 may have greater specificity for a narrower definition of auditory hallucinations.

Future studies should further investigate how MID VH items and SCID B16 relate to one another. We consider possible relationships: 1) VH in trauma-related disorders could be a ‘phenocopy' of VH in psychotic disorders. In the field of genetics, the term phenocopy refers to a phenotype that falsely mimics the true phenotype (129). Phenocopies have a different cause than the condition that they mimic. Using phenocopies in genetics as a metaphor, perhaps VH in trauma-related and schizophrenia spectrum disorders arise from fundamentally different pathogenesis and are only superficially similar (qualitatively or even just semantically). 2) It is possible that psychosis and dissociation share fundamental similarities, and that VH in trauma spectrum and schizophrenia spectrum disorders lie on a common psychosis-dissociation continuum. McCarthy-Jones and Longden (125) have reported, though through indirect comparisons, that the phenomenology of VH in PTSD and schizophrenia is highly similar, and that differences, if they exist, are quantitative rather than qualitative. 3) Lastly, dissociation and psychosis may be phenomena that are distinct but associated with one another. With respect to this latter hypothesis, several researchers have proposed that dissociation operates as a mediator in the relationship between trauma and VH (130–133). An association between dissociation and VH has been described in PTSD (45, 46) and DID (92, 93, 134), as well as in psychosis (131, 132, 135–144). It has been theorized that dissociative detachment, induced by trauma, deprives the individual of both external and internal “anchors,” including the “sense of being connected to one's own body, a sense of self or identity, and one's own actions,” thus leading to impaired reality testing, confusion, disorganization, and disorientation (145). It is possible that VH as measured by the MID, which captures VH from a dissociative framework, may be a mediator between childhood trauma and more psychotic forms of VH.

Despite the significant overlap in symptomatology between trauma spectrum and schizophrenia spectrum disorders, research efforts in the respective fields of trauma-related disorders and schizophrenia have mostly occurred along separate paths (146). Most schizophrenia studies do not measure trauma-related or dissociative symptoms, and studies investigating PTSD and/or dissociative disorders infrequently administer psychosis scales. The current study applies measures of dissociation and psychosis only to individuals with trauma-related disorders. To better answer the question of whether VH in trauma-related disorders is psychotic or even contains components that are psychotic and not just dissociative, studies that directly compare trauma spectrum and schizophrenia spectrum patients using the same “grid” of instruments—including both well-validated dissociation measures and well-validated psychosis measures—are critically needed. Future investigations that employ neuroimaging, electrophysiology, and/or other methods to directly compare the neural mechanisms underlying VH in trauma-related and schizophrenia spectrum disorders may also shed more light into the degree to which VH experiences in these conditions are fundamentally similar or merely phenocopies of one another.

### Current Findings in Relation to Previous Studies

Given the discrepancy of our two estimates of VH rate which are derived from a single patient sample, it is no surprise that such wide variability of estimates exists among previous studies, which collectively applied numerous different methods of defining and measuring VH (**Table 1A****,** **B**). Many of the studies that used standardized dissociation instruments to estimate VH show that the majority of individuals with trauma-related disorders experience VH. For example, Brewin and Patel (45) used the Dissociative Experiences Scale (DES) (63) to assess VH among military veterans with PTSD and found a VH rate of 59.6%. Two independent research groups, Ross and colleagues (56) and Sar and colleagues (8), administered the Dissociative Disorders Interview Schedule (DDIS) (70, 71) and found similarly high rates of individuals who heard voices talking (87.2% and 94.3%, respectively) despite sampling dissociative disorders patients from different countries (USA, Turkey). Boon and Draijer (59) used the Structured Clinical Interview for Dissociative Disorders (SCID-D) (89) to estimate VH in dissociative identity disorder patients from The Netherlands and found that 94.2% reported voices commenting and 90.1% voices arguing. Similarly, Dell (10) administered the MID (the dissociation instrument used in this study) to DID patients and found that 95% reported voices commenting and 89% voices arguing. These estimates, especially the latter which are drawn from samples of patients with dissociative disorders, are in line with the VH rate of 91.4% that we observed in our sample of women with PTSD, many of whom also have dissociative disorders.

Relative to studies that used dissociation-oriented measures, studies employing psychosis-oriented assessment tools have generally reported lower VH rates. For example, two studies used the SCID for DSM-III-R (87) to estimate auditory hallucinations in combat veterans with PTSD: David and colleagues (42) reported a rate of 37.7%, and Hamner and colleagues (43) 46.7%. These estimates are highly consistent with the VH rate of 45.7% that we found using SCID B16 in this study. Of note, the auditory hallucinations item in the SCID for DSM-III-R (B35) is identical to item B16 in the SCID for DSM-IV-TR used in the current study. Butler and colleagues (41), using the Scale for the Assessment of Positive Symptoms (SAPS) (85), estimated that 25% of Vietnam veterans with PTSD related to verifiable combat experience had hallucinations (this study estimated hallucinations in any sensory modality, not auditory hallucinations or VH, per se). Anketell and colleagues (46), using the Positive and Negative Syndrome Scale (PANSS) (64) in combination with specific inquiries about “noises and voices inside their head that others can't hear”, estimated that VH occurred in 50% of chronic PTSD patients. While studies that use psychosis instruments to ascertain VH in trauma-related disorders have generally reported lower rates, it should also be noted that most of the studies that use psychosis-oriented assessment tools were conducted in PTSD patients, while nearly all dissociative disorders studies used instruments from the dissociation literature.

The variability in the literature is not only due to differences in how VH is measured but is also related to differences in patient samples. VH has been reported in 13.9-59.6% of patients with PTSD related to military combat (7, 39–43, 45, 46), and in 5-85% of civilian samples with PTSD (44–46, 48). The rate of VH among refugees with PTSD was 27.1% (47) (**Table 1A**). Several dissociative disorders studies clearly described patient samples in which the majority had been exposed to childhood trauma, and these studies reported VH rates of 30%–100% (**Table 1B**).

To our knowledge, ours is the first study to examine VH in patients with PTSD related to childhood abuse, many of whom also had high levels of dissociative symptoms. Our study sample was generally characterized by a high overall trauma load, including verbal, physical, and sexual abuse afflicted by multiple perpetrators, starting very early in life, and that was experienced recurrently over extended periods of time. Consistent with these features, the vast majority reported severe histories of childhood abuse and neglect across all domains assessed in the CTQ. Further, patients in our sample had high levels of dissociation. For example, the average MID severe dissociation score of the unequivocal voice hearers was 99.6 and that of the ambiguous voice hearers was 85.2. These scores indicate that on average the groups endorsed over half (59% and 51%, respectively) of the pathological dissociation items on the MID to a clinically significant level.

Studies conducted in samples consisting primarily of adult women with DSM-IV or DSM-IV-TR DID—which are the most clinically and demographically comparable to our sample—found rates of VH that are strikingly consistent with our rate of 91.4% as measured by the MID. For example, Sar and colleagues (8) reported a VH rate of 94.3%, Middleton and Butler (9) reported 98%, and Dell (10) 95%. Though Putnam and colleagues (51) appear to have studied a comparable sample of mostly women with dissociative disorders, they reported a much lower VH rate, of 30%. This rate appears to be an outlier among the dissociative disorders studies reviewed. Notably, the Putnam study did not involve any direct participation by patients, but rather utilized a mail-in survey to clinicians, which may be subject to greater clinician bias. Our estimates also contrast starkly with the VH rate of only 5% which Clifford and colleagues (48) recently reported in a civilian sample with PTSD related to physical and sexual abuse. However, in contrast to our sample, the sample in the Clifford study (48) was characterized by markedly less dissociative symptoms, as evidenced by a mean ± SD DES-II score of 26.8 ± 19.0, which falls below the average DES score of 48 for DID in research and even below the average DES score of 31 for PTSD (67). The associations observed in our sample between non-VH and trauma-related diagnosis of PTSD alone (without dissociation) and with lower MID dissociation scores suggest that VH may be closely linked to the experience of dissociative symptoms. Furthermore, while the Clifford study is similar to ours in that they studied a civilian sample with physical and sexual abuse, less than half of their sample had experienced such trauma during childhood. The lower rate of childhood trauma in the Clifford study contrasts with our study, which required participants to have a history of childhood abuse to be study eligible. Lastly, there may be some sex differences in the rate of VH among trauma-related disorders (34, 50), and the gender distribution of the Clifford sample was not specified.

### Limitations

Our findings should be considered in the context of several limitations. First, as we have discussed, our study is limited in its ability to resolve whether the unequivocal voice hearers in our sample (i.e., those who endorsed SCID B16 in addition to MID VH items) experience VH that is not just dissociative but also psychotic in nature. The SCID only screens for lifetime presence or absence of auditory hallucinatory experiences. We did not administer other well-validated psychosis measures, such as the SAPS or PANSS, which are designed to measure differences in symptom severity. Nor did we administer more multidimensional hallucination scales, such as the Psychotic Symptom Rating Scale (PSYRATS) (147) or the Auditory Hallucination Rating Scale (AHRS) (148), which provide more phenomenologically rich information about auditory hallucinations. More importantly, we investigated VH only in trauma spectrum patients. There is a critical need to directly compare trauma and schizophrenia spectrum patients with respect to VH epidemiology, phenomenology, and underlying neurobiology to determine whether the transdiagnostic experience of VH is fundamentally similar or different in these two categories of psychiatric illness.

Second, there are limitations related to the representativeness of our sample: (a) This study entailed secondary analysis of existing data, and the data were not collected from all patients consecutively admitted to or evaluated at our trauma specialty service. Thus, the total sample in our denominator may not be fully representative of individuals with TSD, let alone individuals with TSD at McLean Hospital. On the other hand, as the aims of the original study did not relate to VH, there was no active bias to deliberately seek out voice hearing individuals for recruitment. (b) Another major limitation is that our sample of 70 patients is small, and the proportions we observed in our study are unlikely to reflect the true prevalence of VH, which would be best estimated by large-scale, population-based epidemiological studies. On the other hand, a sample size of 70 is relatively large in the scope of the existing literature on dissociative phenomenology, especially the literature on VH in TSD as can be seen in **Table 1A****,** **B**. (c) We studied a fairly narrow subgroup of TSD—namely, female PTSD patients with childhood trauma histories and severe dissociative symptoms who were receiving care at a specialized PTSD treatment center. Thus, our findings may not be broadly generalizable. On the other hand, most existing studies of VH in PTSD were conducted in primarily male combat veterans. Women, especially those with histories of childhood abuse, are relatively underrepresented in PTSD research, and our study helps to fill a gap in the literature about the rate and phenomenology of VH in women with PTSD and dissociative psychopathology related to childhood trauma. Another important point is that while our small and somewhat unique sample might limit the generalizability of our findings, issues relating to the representativeness of our sample do not take away from one of our main findings that even within a single sample, two different instruments lead to two very different estimates of VH. d) We combined PTSD patients with and without dissociative symptoms into a single TSD group. However, our results (**Table 3**) suggest that these groups may differ in the distribution of VH experiences. The rates of VH observed in our sample may better approximate the prevalence of VH in individuals who suffer a constellation of both PTSD and dissociative symptoms, and likely overestimate the rate of VH in PTSD samples with minimal dissociative psychopathology.

Third, the frequency and experience of VH within an individual can change over time, and our data, which are cross-sectional, are limited in the ability to provide insights about the evolution or stability of VH over time. More longitudinal studies are needed to address this issue. Fourth, a related consideration is that clinical acuity could potentially impact the frequency and severity of VH, and our sample consisted of patients at various levels of care, including outpatient, partial hospital, residential, and inpatient settings. However, the unequivocal and ambiguous VH groups did not statistically significantly differ on measures of PTSD, dissociation, or depressive symptom severity.

Fifth, all participants were medicated at the time of study participation, and it is unclear what effect medications, especially antipsychotic medications, had on the experience of VH in this sample. Two small double-blind, randomized placebo-controlled trials provide preliminary evidence that the antipsychotic risperidone may be effective in women with chronic non-combat related PTSD (149, 150). However, other studies have not demonstrated sufficient evidence of benefit of antipsychotic medications in the treatment of PTSD (151) or DID (152), and, to our knowledge, there have been no studies systematically investigating the effect of antipsychotic medications specifically on VH in trauma-related disorders. Even if antipsychotic medications did have the effect of reducing VH, there was no statistically significant difference in the proportion of patients in our sample who were on antipsychotic medications across the three groups. The unequivocal non-VH (B16-/MID-) group did contain a higher proportion of patients on antipsychotic medications (83.3%) relative to the other two groups (43.8% in the unequivocal VH group, 34.4% in the ambiguous VH group), even if this difference was not statistically significant. Thus, some could question if the absence of VH in this group could be related to the greater proportion of patients being treated with antipsychotic medications in this group. For example, it is conceivable that antipsychotics could have been prescribed because of VH. However, this scenario is unlikely since the SCID item B16 asks about ever/lifetime history of VH or auditory hallucinations (not just current or recent experiences), and this item was coded as negative for all patients in this group. More importantly, the two VH groups for whom we make phenomenological comparisons were more comparable with respect to antipsychotic exposure. Therefore, the phenomenological differences we found between the two VH groups do not appear to be due to any systematic differences in antipsychotic exposure between them.

Finally, it is worth acknowledging that any attempt to study and phenomenologically classify a subjective experience, including VH, is inevitably fraught with semantic challenges. Language is a necessary vehicle for communication but can be limited in its ability to accurately reflect the nuances of an individual's internal experience. People may not always have the words to describe the subtleties and complexities of subjective self-experiences, which unlike other things in everyday experience have no physical characteristics (153). This may be especially true when it comes to more unusual internal experiences, which may have an ineffable and pre-reflective quality (154, 155). Moreover, depending on an individual's life experience and background, the same words can have different meanings for different people. This is true not only for patients, but also for clinicians and researchers in different fields who may have differing theoretical frameworks and expertise (153). Neurobiological approaches to investigating similarities and differences between “psychotic” vs. “dissociative” vs. other types of VH may help to move past some of these semantic barriers.

### Conclusions

The experience of VH is given little diagnostic or treatment consideration in trauma spectrum disorders. There is little to no mention of VH in previous or current versions of the DSM sections on PTSD and dissociative disorders. However, our estimates of VH rate (45.7% using SCID B16, and 91.4% using MID VH items) are in line with previous studies that used comparable instruments and comparable patient samples, and suggest that VH is more ubiquitous in trauma-related disorders than traditionally recognized. We found high rates of VH in our sample of women with trauma spectrum disorders, irrespective of method of inquiry. However, the MID-based rate of 91.4%, in particular, suggests that VH may not merely be a secondary feature of trauma-related disorders, but rather reflective of some core process or phenomenon more fundamental in posttraumatic and especially dissociative conditions. It remains to be resolved whether VH in trauma-related disorders has components that are psychotic in addition to dissociative in a subset of patients. Nonetheless, our results add to the growing scientific evidence that hearing voices—which is an experience typically considered psychotic or psychotic-like—is not equivalent to having a psychotic disorder. In fact, it is increasingly recognized that there is a continuum of VH that transcends even the boundaries of mental illness, extending to individuals with VH in the general population (18, 156). Increased recognition among both patients and clinicians that VH is not uncommon in trauma-related disorders can reduce potential misdiagnosis of PTSD and dissociative disorder as cases of schizophrenia, which in turn has significant treatment implications.

A key finding from our study is that it matters how you ask about VH. Using only instruments that are narrowly designed to detect VH and other psychotic symptoms in schizophrenia and related psychotic disorders may potentially have greater specificity for a certain form of VH, but underestimate a broader range of VH experiences. Until we have better understanding about the underlying nature and pathogenesis of different types of VH, instruments that assess VH more agnostically, apart from psychotic disorders, and that capture the multidimensional nature of the experience should be considered to improve identification of VH experiences, especially among patients with non-psychotic disorders.

## Data Availability Statement

The datasets generated for this study are available on request to the corresponding author.

## Ethics Statement

The study was approved by the Partners Healthcare Institutional Review Board. All participants gave written informed consent in accordance with the Declaration of Helsinki.

## Author Contributions

AS analyzed the data, interpreted the results, and drafted the manuscript. MH analyzed the data and contributed to results interpretation and manuscript drafting. MK, JW, and LL collected the data and contributed to interpretation of the results and manuscript drafting. SW contributed to data collection and interpretation. MR contributed to data analysis and interpretation of the results. DO and KR contributed to interpretation of the results and manuscript drafting. All authors approved the final manuscript version.

## Funding

This research was supported by NIH K23MH100611 and the McLean Hospital Pope-Hintz Fellowship to AS, and NIH R21MH112986 to MK and KR.

## Conflict of Interest

The authors declare that the research was conducted in the absence of any commercial or financial relationships that could be construed as a potential conflict of interest.
