# Supplementary material for: Assessing Voice Hearing in Trauma Spectrum Disorders: A Comparison of Two Measures and a Review of the Literature
Source: Front Psychiatry. 2020 Feb 24;10:1011. doi: 10.3389/fpsyt.2019.01011 (PMC7050446; doi:10.3389/fpsyt.2019.01011)
Supplement: Supplementary file 1 [file DataSheet_1.pdf]

Supplementary Table 1. Inventory of Childhood Abuse

Type of Childhood Abuse		All	Unequivocal VH + (B16 +/ MID +)	Ambiguous VH + (B16 – / MID +)	Unequivocal VH – (B16 – / MID –)	Statistic	Significance
		N=70	N=32	N=32	N=6		
**Verbally abused as a child**, No. (%)	Yes	48 (69%)	20 (71%)	22 (85%)	6 (100%)	$\chi^2 = 3.132$	$p = 0.209$
	No	12 (17%)	8 (29%)	4 (15%)	0 (0%)		
	Missing	*4 (6%)*					
**Beaten or physically punished (excluding spanking) as a child**, No. (%)	Yes	45 (64%)	20 (67%)	21 (70%)	4 (67%)	$\chi^2 = 0.084$	$p = 0.959$
	No	21 (30%)	10 (33%)	9 (30%)	2 (32%)		
	Missing	*4 (6%)*					
**Attacked with a gun/knife/other weapon by intimate partner at age < 18y**, No. (%)†	Yes	1 (1%)	0 (0%)	1 (3%)	0 (0%)	-	-
	No	63 (90%)	30 (100%)	27 (97%)	6 (100%)		
	Missing	*6 (9%)*					
**Attacked with a gun/knife/other weapon by someone other than intimate partner < 18y**, No. (%)†	Yes	17 (24%)	8 (27%)	8 (27%)	1 (17%)	$\chi^2 = 0.285$	$p = 0.867$
	No	49 (70%)	22 (73%)	22 (73%)	5 (83%)		
	Missing	*4 (6%)*					
**Attacked without a weapon by intimate partner at age < 18y**, No. (%)††	Yes	7 (10%)	2 (7%)	5 (17%)	0 (0%)	$\chi^2 = 2.514$	$p = 0.285$
	No	58 (83%)	28 (93%)	24 (83%)	6 (100%)		
	Missing	*5 (7%)*					
**Attacked without a weapon by someone other than intimate partner at age < 18y**, No. (%)††	Yes	38 (54%)	18 (60%)	17 (57%)	3 (50%)	$\chi^2 = 0.223$	$p = 0.894$
	No	28 (40%)	12 (40%)	13 (43%)	3 (50%)		
	Missing	*4 (6%)*					
**Sexually abused between ages 0-13y**, No. (%)†††	Yes	54 (77%)	26 (90%)	24 (80%)	4 (67%)	$\chi^2 = 2.244$	$p = 0.326$
	No	11 (16%)	3 (10%)	6 (20%)	2 (33%)		
	Missing	*5 (7%)*					
**Sexually abused between ages 14-17y**, No. (%)†††	Yes	34 (49%)	18 (64%)	14 (47%)	2 (33%)	$\chi^2 = 2.847$	$p = 0.241$
	No	30 (43%)	10 (36%)	16 (53%)	4 (67%)		
	Missing	*6 (9%)*					
**Total number, types of childhood abuse,** mean ± SD (range)	*Missing in n=4*	3.7 ± 1.5 (1-7)	3.7 ± 1.4 (1-6)	3.7 ± 1.6 (1-7)	3.3 ± 1.6 (1-5)	$F = 0.182$	$p = 0.834$

† Total number of patients with a history of attack with a gun/knife/other weapon in childhood irrespective of whether attacked by intimate partner or other is n=18 (8 unequivocal VH+, 9 ambiguous VH+, 1 unequivocal VH-).

†† Total number of patients with a history of attack without a weapon in childhood irrespective of whether attacked by intimate partner or other is n=43 (19 unequivocal VH+, 21 ambiguous VH+, 3 unequivocal VH-).

††† Total number of patients with any sexual abuse in childhood (ages 0-13 and ages 14-17 combined) is n=57 (28 unequivocal VH+, 24 ambiguous VH+, 5 unequivocal VH-).

Supplementary Table 2. Average First Ages and Durations of Childhood Abuse

Type of Childhood Abuse	All	Unequivocal VH + (B16 +/ MID +)	Ambiguous VH + (B16 – / MID +)	Unequivocal VH – (B16 – / MID –)	Statistic	Significance
**Verbally abused as a child**						
**First age**, y, mean ± SD (range)[1]	5.3 ± 3.3 (1-15) (N=46)	5.3 ± 2.9 (1-15) (n=18)	5.3 ± 3.9 (1-15) (n=22)	5.0 ± 1.8 (3-8) (n=6)	*F* = 0.022	*p* = 0.978
**Duration**, y, mean ± SD (range)[2]	16.5 ± 10.1 (1-53) (N=42)	17.6 ± 12.1 (3-53) (n=15)	15.5 ± 9.6 (1-45) (n=21)	17.3 ± 6.9 (9-26) (n=6)	*F* = 0.208	*p* = 0.813
**Beaten or physically punished as a child**						
**First age**, y, mean ± SD (range)[3]	5.1 ± 2.7 (2-14) (N= 41)	4.7 ± 1.6 (2-8) (n=17)	4.5 ± 2.1 (2-10) (n=20)	9.5 ± 5.3 (4-14) (n=4)	*F* = 7.988	***p* = 0.001***
**Duration**, y, mean ± SD (range)[4]	10.8 ± 5.6 (0-25) (N=39)	12.0 ± 5.9 (3-25) (n=16)	10.2 ± 4.9 (0-16) (n=19)	9.0 ± 8.4 (2-19) (n=4)	*F* = 0.680	*p* = 0.513
**Attacked with a gun/knife/other weapon by intimate partner at age < 18y**						
**First age**, y, mean ± SD (range)	13 (N=1)	-	-	-	-	-
**Duration**, y, mean ± SD (range)†	-	-	-	-	-	-
**Attacked with gun/knife/other weapon by other than intimate partner at age < 18y**						
**First age**, y, mean ± SD (range)[1]	8.1 ± 3.8 (3-14) (N=17)	10.0 ± 2.8 (5-14) (n=8)	6.6 ± 4.1 (3-14) (n=8)	5 (n=1)	*F* = 2.250	*p* = 0.142
**Duration**, y, mean ± SD (range)†	-	-	-	-	-	-
**Attacked without a weapon by intimate partner at age < 18y**						
**First age**, y, mean ± SD (range)[1]	14.3 ± 2.6 (10-17) (N=7)	15.0 ± 2.8 (13-17) (n=2)	14.0 ± 2.7 (10-17) (n=5)	- (n=0)	*F* = 0.188	*p* = 0.683
**Duration**, y, mean ± SD (range)†	-	-	-	-	-	-
**Attacked without a weapon by someone other than intimate partner at age < 18y**						
**First age**, y, mean ± SD (range)[1]	6.9 ± 4.1 (2-16) (N=38)	7.0 ± 4.0 (2-15) (n=18)	6.6 ± 4.2 (2-16) (n=17)	8.0 ± 5.3 (4-14) (n=3)	*F* = 0.141	*p* = 0.869
**Duration**, y, mean ± SD (range)†	-	-	-	-	-	-
**Sexually abused as a child (ages 0-17y)**						
**First age**, y, mean ± SD (range)[5]	5.6 ± 3.3 (2-17) (N=50)	6.0 ± 3.6 (3-15) (n=24)	4.8 ± 1.9 (2-9) (n=22)	7.8 ± 6.2 (4-17) (n=4)	*F* = 1.729	*p* = 0.189
**Duration**, y, mean ± SD (range)[6]	8.0 ± 5.1 (0-21) (N=46)	8.3 ± 6.2 (0-21) (n=21)	8.3 ± 4.1 (0-16) (n=21)	4.3 ± 3.3 (0-8) (n=4)	*F* = 1.156	*p* = 0.324

[1] Info about first age of verbal abuse missing in 2 of 48 patients (2 unequivocal VH+) who reported childhood verbal abuse.
[2] Info about duration of verbal abuse missing in 6 of 48 patients (5 unequivocal VH+, 1 ambiguous VH+) who reported childhood verbal abuse.
[3] Info about first age of physical abuse missing in 4 of 45 patients (3 unequivocal VH+, 1 ambiguous VH+) who reported childhood physical abuse.
[4] Info about duration of physical abuse missing in 6 of 45 patients (4 unequivocal VH+, 2 ambiguous VH+) who reported childhood physical abuse.

[5] Info about first age of sexual abuse missing in 7 of 57 patients (4 unequivocal VH+, 2 ambiguous VH+, 1 unequivocal VH-) who reported sexual abuse in ages 0-17 years.
[6] Info about duration of sexual abuse missing in 11 of 57 patients (7 unequivocal VH+, 3 ambiguous VH+, 1 unequivocal VH-) who reported sexual abuse in ages 0-17 years.
† The Traumatic Events Inventory (TEI) does not assess the last age at which a patient experienced an attack with or without a weapon, thus preventing calculation of duration of exposure for these events.
*Post-hoc comparisons using Tukey's test for multiple comparisons correction shows statistically significant differences between the unequivocal VH- group and the unequivocal VH+ group (p = 0.002), and between the unequivocal VH- group and the ambiguous VH+ group (p = 0.001).

Supplementary Table 3. Perpetrators of Verbal, Physical, or Sexual Abuse in Childhood

Perpetrator	All	Unequivocal VH + (B16 +/ MID +)	Ambiguous VH + (B16 – / MID +)	Unequivocal VH – (B16 – / MID –)	Statistic	Significance
	N=65†	N=30†	N=29†	N=6		
**Primary caregiver**, No. (%)	55 (85%)	24 (80%)	25 (86%)	6 (100%)	$\chi^2 = 1.638$	$p = 0.441$
Mother/female primary caregiver	40 (62%)	17 (57%)	18 (62%)	5 (83%)	$\chi^2 = 1.508$	$p = 0.470$
Father/male primary caregiver	38 (59%)	17 (57%)	17 (59%)	4 (67%)	$\chi^2 = 0.206$	$p = 0.902$
**Other family, non-primary caregivers**, No. (%)	41 (63%)	18 (60%)	19 (66%)	4 (67%)	$\chi^2 = 0.229$	$p = 0.892$
Stepparent or Partner of Parent	8 (12%)	4 (13%)	3 (10%)	1 (17%)	$\chi^2 = 0.238$	$p = 0.888$
Other adult family	26 (40%)	11 (37%)	11 (38%)	4 (67%)	$\chi^2 = 1.968$	$p = 0.374$
Older sibling	18 (28%)	8 (27%)	10 (35%)	0 (0%)	$\chi^2 = 2.981$	$p = 0.225$
**Non-family**, No. (%)	42 (65%)	20 (67%)	19 (66%)	3 (50%)	$\chi^2 = 0.626$	$p = 0.731$
Other adult, known	24 (37%)	11 (37%)	11 (38%)	2 (33%)	$\chi^2 = 0.047$	$p = 0.977$
Other adult, stranger	17 (26%)	9 (30%)	7 (24%)	1 (17%)	$\chi^2 = 0.570$	$p = 0.752$
Other	20 (31%)	6 (20%)	12 (41%)	2 (33%)	$\chi^2 = 3.184$	$p = 0.203$
**Total number of abusers during childhood**, mean ± SD (range)	2.9 ± 1.5 (1-7)	2.8 ± 1.3 (1-5)	3.1 ± 1.8 (1-7)	3.2 ± 1.2 (2-5)	$F = 0.369$	$p = 0.693$

†N's reflect the number for whom TEI data on perpetrators of childhood verbal abuse, physical beatings, and sexual abuse are available. This information is not applicable for one patient (ambiguous VH+) who reported no childhood verbal abuse, physical beatings, or sexual abuse; this patient reported a history of being attacked without a weapon by someone other than intimate partner at age 16. The TEI does not systematically inquire about specific perpetrators for questions related to history of being attacked. Data are missing for four of the 70 total patients (2 unequivocal VH+, 2 ambiguous VH+).

Supplementary Table 4. Traumatic Events Inventory for Lifetime Trauma

Traumatic Event		All	Unequivocal VH + (B16 +/ MID +)	Ambiguous VH + (B16 – / MID +)	Unequivocal VH – (B16 – / MID –)	Statistic	Significance
		N=70	N=32	N=32	N=6		
**Witnessed violence between parents as a child**, No. (%)	Yes	37 (53%)	16 (53%)	19 (63%)	2 (33%)	χ^2 = 1.993	*p* = 0.369
	No	29 (41%)	14 (47%)	11 (37%)	4 (67%)		
	Missing	*4 (6%)*					
**Verbally abused as a child**, No. (%)	Yes	48 (69%)	20 (71%)	22 (85%)	6 (100%)	χ^2 = 3.132	*p* = 0.209
	No	12 (17%)	8 (29%)	4 (15%)	0 (0%)		
	Missing	*4 (6%)*					
**Beaten or physically punished (excluding spanking) as a child**, No. (%)	Yes	45 (64%)	20 (67%)	21 (70%)	4 (67%)	χ^2 = 0.084	*p* = 0.959
	No	21 (30%)	10 (33%)	9 (30%)	2 (32%)		
	Missing	*4 (6%)*					
**Sexually abused between ages 0-13y**, No. (%)†	Yes	54 (77%)	26 (90%)	24 (80%)	4 (67%)	χ^2 = 2.244	*p* = 0.326
	No	11 (16%)	3 (10%)	6 (20%)	2 (33%)		
	Missing	*5 (7%)*					
**Sexually abused between ages 14-17y**, No. (%)†	Yes	34 (49%)	18 (64%)	14 (47%)	2 (33%)	χ^2 = 2.847	*p* = 0.241
	No	30 (43%)	10 (36%)	16 (53%)	4 (67%)		
	Missing	*6 (9%)*					
**Raped or sexually assaulted after age 17**, No. (%)	Yes	38 (54%)	18 (67%)	16 (62%)	4 (67%)	χ^2 = 0.167	*p* = 0.920
	No	21 (30%)	9 (33%)	10 (39%)	2 (33%)		
	Missing	*11 (16%)*					
**Witnessed murder of close friend or family**, No. (%)	Yes	1 (1%)	0 (0%)	1 (3%)	0 (0%)	χ^2 = 1.218	*p* = 0.544
	No	65 (93%)	30 (100%)	29 (97%)	6 (100%)		
	Missing	*4 (6%)*					
**Confronted with knowledge of murder of close friend or family**, No. (%)	Yes	11 (16%)	3 (10%)	7 (23%)	1 (17%)	χ^2 = 1.920	*p* = 0.383
	No	55 (79%)	27 (90%)	23 (77%)	5 (83%)		
	Missing	*4 (6%)*					
**Attacked with a gun/knife/other weapon by intimate partner**, No. (%)	Yes	6 (9%)	3 (10%)	2 (7%)	1 (17%)	χ^2 = 0.553	*p* = 0.758
	No	58 (83%)	27 (90%)	26 (93%)	5 (83%)		
	Missing	*6 (9%)*					
**Attacked with a gun/knife/other weapon by someone other than intimate partner**, No. (%)	Yes	30 (43%)	15 (50%)	13 (43%)	2 (33%)	χ^2 = 0.660	*p* = 0.719
	No	36 (51%)	15 (50%)	17 (57%)	4 (67%)		
	Missing	*4 (6%)*					
**Witnessed family/friend attacked with a gun/knife/other weapon**, No. (%)	Yes	17 (24%)	4 (14%)	11 (38%)	2 (33%)	χ^2 = 4.487	*p* = 0.106
	No	47 (67%)	25 (87%)	18 (62%)	4 (67%)		
	Missing	*6 (9%)*					

**Witnessed someone other than family/friend attacked with a gun/knife/other weapon**, No. (%)	Yes	13 (19%)	6 (20%)	6 (21%)	1 (20%)	$\chi^2 = 0.005$	$p = 0.998$
	No	51 (73%)	24 (80%)	23 (79%)	4 (80%)		
	Missing	*6 (9%)*					
**Attacked without a weapon by intimate partner**, No. (%)	Yes	25 (36%)	10 (33%)	14 (48%)	1 (17%)	$\chi^2 = 2.718$	$p = 0.257$
	No	40 (57%)	20 (67%)	15 (52%)	5 (83%)		
	Missing	*5 (7%)*					
**Attacked without a weapon by someone other than intimate partner**, No. (%)	Yes	49 (70%)	25 (83%)	19 (63%)	5 (83%)	$\chi^2 = 3.423$	$p = 0.181$
	No	17 (24%)	5 (17%)	11 (37%)	1 (17%)		
	Missing	*4 (6%)*					
**Witnessed family/friend attacked without a weapon**, No. (%)	Yes	38 (54%)	13 (43%)	20 (67%)	5 (83%)	$\chi^2 = 5.136$	$p = 0.077$
	No	28 (40%)	17 (57%)	10 (33%)	1 (17%)		
	Missing	*4 (6%)*					
**Witnessed someone other than family/friend attacked without a weapon**, No. (%)	Yes	21 (30%)	7 (25%)	11 (38%)	3 (50%)	$\chi^2 = 1.901$	$p = 0.387$
	No	42 (60%)	21 (75%)	18 (62%)	3 (50%)		
	Missing	*7 (10%)*					
**Experienced a natural disaster**, No. (%)	Yes	20 (29%)	11 (37%)	7 (23%)	2 (33%)	$\chi^2 = 1.483$	$p = 0.476$
	No	47 (67%)	19 (63%)	24 (78%)	4 (67%)		
	Missing	*3 (3%)*					
**Experienced a serious accident or injury**, No. (%)	Yes	27 (39%)	11 (38%)	13 (42%)	3 (50%)	$\chi^2 = 0.325$	$p = 0.850$
	No	39 (56%)	18 (62%)	18 (58%)	3 (50%)		
	Missing	*4 (6%)*					
**Witnessed a serious accident or injury**, No. (%)	Yes	37 (53%)	15 (50%)	18 (58%)	4 (67%)	$\chi^2 = 0.750$	$p = 0.687$
	No	30 (43%)	15 (50%)	13 (42%)	2 (33%)		
	Missing	*3 (4%)*					
**Experienced a sudden life-threatening illness**, No. (%)	Yes	12 (17%)	6 (20%)	6 (19%)	0 (0%)	$\chi^2 = 1.442$	$p = 0.486$
	No	55 (79%)	24 (80%)	25 (81%)	6 (100%)		
	Missing	*3 (4%)*					
**Been in military combat/service in a war zone**, No. (%)	Yes	0 (0%)	0 (0%)	0 (0%)	0 (0%)	-	-
	No	66 (94%)	29 (100%)	31 (100%)	6 (100%)		
	Missing	*4 (6%)*					
**Total number of lifetime traumas**, mean ± SD (range)	*Missing in n=3*	8.4 ± 3.6 (2-18)	8.23 ± 3.3 (2-16)	8.5 ± 3.9 (2-18)	8.7 ± 3.2 (6-15)	$F = 0.065$	$p = 0.937$
